# A new flexible Weibull extension model: Different estimation methods and modeling an extreme value data

**DOI:** 10.1016/j.heliyon.2023.e21704

**Published:** 2023-10-31

**Authors:** Huda M. Alshanbari, Omalsad Hamood Odhah, Hazem Al-Mofleh, Zubair Ahmad, Saima K. Khosa, Abd al-Aziz Hosni El-Bagoury

**Affiliations:** a Department of Mathematical Sciences, College of Science, Princess Nourah bint Abdulrahman University, P.O. Box 84428, Riyadh 11671, Saudi Arabia; b Division of Science and Mathematics, Eureka College, Eureka, IL 61530, USA; c Department of Mathematics, Tafila Technical University, Tafila 66110, Jordan; d Department of Statistics, Quaid-i-Azam University, Islamabad 44000, Pakistan; e Department of Mathematics and Statistics, University of Saskatchewan, Saskatoon, SK, Canada; f Basic Sciences Department, Higher Institute of Engineering and Technology, EL-Mahala EL-Kobra, Egypt

**Keywords:** Flexible Weibull extension distribution, Extreme value theory, Distributional properties, Estimation, Flood data

## Abstract

The word extreme events refer to unnatural or undesirable events. Due to the general destructive effects on society and scientific problems in various applied fields, the study of extreme events is an important subject for researchers. Many real-life phenomena exhibit clusters of extreme observations that cannot be adequately predicted and modeled by the traditional distributions. Therefore, we need new flexible probability distributions that are useful in modeling extreme-value data in various fields such as the financial sector, telecommunications, hydrology, engineering, and meteorology. In this piece of research work, a new flexible probability distribution is introduced, which is attained by joining together the flexible Weibull distribution with the weighted T-*X* strategy. The new model is named a new flexible Weibull extension distribution. The distributional properties of the new model are derived. Furthermore, some frequently implemented estimation approaches are considered to obtain the estimators of the new flexible Weibull extension model. Finally, we demonstrate the utility of the new flexible Weibull extension distribution by analyzing an extreme value data set.

## 1 Introduction

A number of probability methodologies (or probability distributions) have been presented and implemented to examine the main body of data and describe its behavior. These probability distributions are effectively implemented for analyzing the crises in the global financial sector, radical political events, military strikes, or natural disasters such as earthquakes, floods, and droughts [1], [2], [3], [4], [5].

However, it is also a proven and confirmed fact that no single probability distribution is apt to provide a satisfactory fit in all circumstances. Therefore, to provide a satisfactory fit to real-life scenarios, we often need new probability distributions with better and improved distributional flexibility. The needs of data modeling with satisfactory fit have motivated researchers to discover and apply new flexible probability distributions [6], [7], [8], [9].

Considering the significance of the probability distributions in modeling the real-life events, numerous probability distributions have been suggested and implemented by improving the existing distributions. The modifications of the existing probability distributions are based on different methods.

The compounding of the probability distributions is a useful approach for developing new probability distributions. However, the new probability models obtained using the compounding approach have a difficult form of density which makes the estimation process more burdensome [10]. The transformation approach, especially, the exponential transformation, is also an effective approach to obtain new models. This approach is one of the easiest methods to use for obtaining new probability distributions. However, the derivation of properties of such distributions becomes very difficult [11]. Another prominent method for obtaining new distributions which provides reasonably the best-suited fit to real-life events is the finite mixture of probability distributions. This method generates new updated probability distributions with improved distributional flexibility. However, the number of parameters of the new models is also increasing. Therefore, more computational efforts and time are needed for deriving the estimators [12].

Here, we study a new probability distribution for dealing with the flood data set. The new model is introduced without incorporating new additional parameters to reduce the estimation problems and has a closed-form CDF. The new model is termed as a new flexible Weibull extension (NFWE) distribution. Despite adding no additional parameter, the NFWE distribution still performs better.

Let U(UR) have the flexible Weibull extension (FWE) distribution [13], its CDF is

(1) $$F(u;\Psi )=1-e^{-e^{\delta_{1}u-\delta_{2}/u}},\;\;\;\;\;u\geq 0,$$

where $\Psi =(\delta_{1},\delta_{1})$, and $\delta_{1},\delta_{1}>0$ are parameters of the FWE distribution. The FWE model is further generalized and implemented by many researchers [14], [15], [16], [17].

The PDF (probability density function) f(u;Ψ), hazard rate function (HRF) h(u;Ψ), as well as the survival function (SF) S(u;Ψ) of the FWE distribution are

$$f(u;\Psi )=\left(\delta_{1}+\frac{\delta_{2}}{u^{2}}\right)e^{\delta_{1}u-\delta_{2}/u}e^{-e^{\delta_{1}u-\delta_{2}/u}},\;\;\;\;\;u>0,$$

$$h(u;\Psi )=\left(\delta_{1}+\frac{\delta_{2}}{u^{2}}\right)e^{\delta_{1}u-\delta_{2}/u},\;\;\;\;\;u>0,$$

and

$$S(u;\Psi )=e^{-e^{\delta_{1}u-\delta_{2}/u}},\;\;\;\;\;u>0,$$

respectively.

The proposed modification of the FWE distribution (i.e., NFWE distribution) is introduced by joining together the FWE model with the weighted T-*X* strategy of [18]. The weighted T-*X* strategy was introduced by using the T-*X* generator [19]. Its CDF, say G(u;Ψ), is given by

(2) $$G(u;\Psi )=1-\frac{\overset{¯}{F}(u;\Psi )}{e^{F(u;\Psi )}},\;\;\;\;\;u\in R,$$

where $\overset{¯}{F}(u;\Psi )=1-F(u;\Psi )$.

Corresponding to Eq. (2), the PDF g(u;Ψ), HRF h(u;Ψ), and SF S(u;Ψ) of the NFWE distribution are given by

$$g(u;\Psi )=\frac{f(u;\Psi )}{e^{F(u;\Psi )}}\left[2-F(u;\Psi )\right],\;\;\;\;\;u\in R,$$

$$h(u;\Psi )=\frac{f(u;\Psi )}{\overset{¯}{F}(u;\Psi )}\left[2-F(u;\Psi )\right],\;\;\;\;\;u\in R,$$

and

$$S(u;\Psi )=\frac{\overset{¯}{F}(u;\Psi )}{e^{F(u;\Psi )}},\;\;\;\;\;u\in R,$$

respectively.

The basic distributional functions of the proposed distribution are provided in Section 2. Furthermore, the graphs of the distributional functions of the NFWE distribution are also given in Section 2.

## 2 A new flexible Weibull extension model

This section introduces the basic functions of the NFWE distribution. By joining Eq. (1) with Eq. (2), we get the CDF of the NFWE distribution as expressed by

(3) $$G(u;\Psi )=1-\frac{e^{-e^{\delta_{1}u-\delta_{2}/u}}}{\exp\left\{1-e^{-e^{\delta_{1}u-\delta_{2}/u}}\right\}},\;\;\;\;\;u\geq 0,$$

with PDF

(4) $$g(u;\Psi )=\frac{\left(\delta_{1}+\frac{\delta_{2}}{u^{2}}\right)e^{\delta_{1}u-\delta_{2}/u}e^{-e^{\delta_{1}u-\delta_{2}/u}}}{\exp\left\{1-e^{-e^{\delta_{1}u-\delta_{2}/u}}\right\}}\left[1+e^{-e^{\delta_{1}u-\delta_{2}/u}}\right],\;\;\;\;\;u>0,$$

where $\Psi ={\left(\delta_{1},\delta_{2}\right)}^{⊺}$.

**Figure 1:**
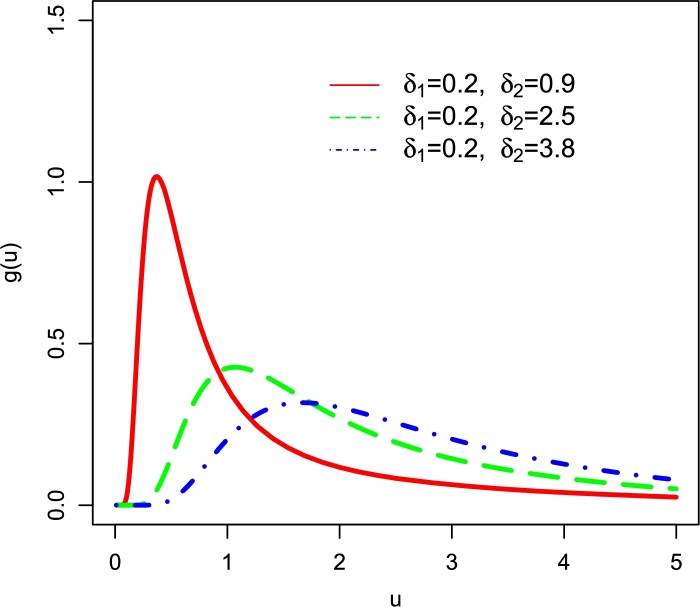
Different PDF plots of the NFWE distribution.

Some behaviors of g(u;Ψ) of the NFWE distribution are shown in Fig. 1, which show that as we increase $\delta_{2}$, the NFWE distribution tends to exhibit a longer tail. This fact reveals that due to the long right tail, the proposed NFWE distribution can be a good candidate model for dealing with the skewed data in telecommunications, finance sector, hydrology, engineering, meteorology, etc.

Furthermore, corresponding to Eq. (3) and Eq. (4), the SF S(u;Ψ)=1−G(u;Ψ) and HF $h(u;\Psi )=\frac{g(u;\Psi )}{S(u;\Psi )}$, are given by

$$S(u;\Psi )=\frac{e^{-e^{\delta_{1}u-\delta_{2}/u}}}{\exp\left\{1-e^{-e^{\delta_{1}u-\delta_{2}/u}}\right\}},\;\;\;\;\;u>0,$$

and

$$h(u;\Psi )=\left(\delta_{1}+\frac{\delta_{2}}{u^{2}}\right)e^{\delta_{1}u-\delta_{2}/u}\left[1+e^{-e^{\delta_{1}u-\delta_{2}/u}}\right],\;\;\;\;\;u>0,$$

respectively.

**Figure 2:**
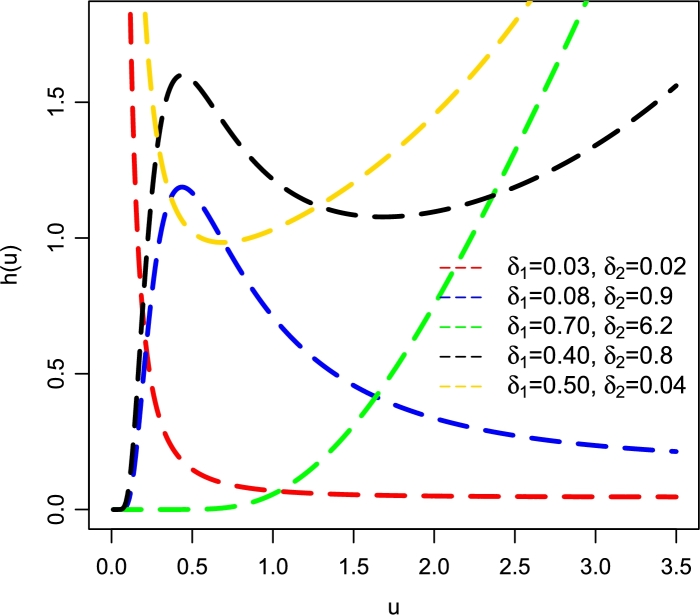
Different HF plots of the NFWE distribution.

Some plots of h(u;Ψ) of the NFWE model are given in Fig. 2, which shows that h(u;Ψ) of the NFWE distribution is capable of capturing five different patterns of the HF such as (*i*) decreasing (also called reverse J-shaped); see red line, (*ii*) unimodal (also called upside down bathtub); see blue line, (*iii*) increasing (or J-shaped); see green line, (*iv*) modified unimodal (black line), and (*v*) bathtub-shaped (gold line).

## 3 Distributional characteristics

Here, we provide a concise mathematical framework of the distributional characteristics of the NFWE distribution. These distributional characteristics involve the quantile function (QF), median, quartiles, skewness, kurtosis, and $r^{th}$ moment.

### 3.1 The quantile function, median, and other quartile measures

The QF of the NFWE distribution, say Q(p), where 0<p<1, is given by

(5) $$Q(p)=\frac{S+\sqrt{4\text{δ}\text{1}\text{δ}\text{2}+S^{2}}}{2\text{δ}\text{1}},$$

where $S=\log(W_{0}((1-p)e)-\log(1-p)-1)$ and $W_{0}(\cdot )$ is the principal Lambert function.

The median of the NFWE model can be obtained using $p=\frac{1}{2}$ in Eq. (5), given by

$$Q\left(\frac{1}{2}\right)=\frac{S+\sqrt{4\text{δ}\text{1}\text{δ}\text{2}+S^{2}}}{2\text{δ}\text{1}},$$

where $S=\log\left(W_{0}\left(\left[1-\frac{1}{2}\right]e\right)-\log\left(1-\frac{1}{2}\right)-1\right)$.

The first quartile (usually expressed by $Q_{1}$) of the NFWE distribution is

$$Q\left(\frac{1}{4}\right)=\frac{S+\sqrt{4\text{δ}\text{1}\text{δ}\text{2}+S^{2}}}{2\text{δ}\text{1}},$$

where $S=\log\left(W_{0}\left(\left[1-\frac{1}{4}\right]e\right)-\log\left(1-\frac{1}{4}\right)-1\right)$.

The third quartile (usually expressed by $Q_{3}$) of the NFWE distribution is

$$Q\left(\frac{3}{4}\right)=\frac{S+\sqrt{4\text{δ}\text{1}\text{δ}\text{2}+S^{2}}}{2\text{δ}\text{1}},$$

where $S=\log\left(W_{0}\left(\left[1-\frac{3}{4}\right]e\right)-\log\left(1-\frac{3}{4}\right)-1\right)$.

The skewness (the Galton's skewness (GS)) of the NFWE distribution can be derived as follows

(6) $$GS=\frac{Q_{6/8}-2Q_{4/8}+Q_{2/8}}{Q_{6/8}-Q_{2/8}},$$

where the quantities $Q_{6/8},Q_{4/8}$, and $Q_{2/8}$ in Eq. (6) can be obtained by using $p=\frac{6}{8},\frac{4}{8}$, and $p=\frac{2}{8}$ in Eq. (5), respectively.

The kurtosis (the Moor's kurtosis (MK)) of the NFWE distribution can be derived as follows

(7) $$MK=\frac{Q_{7/8}-Q_{5/8}-Q_{1/8}+Q_{3/8}}{Q_{6/8}-Q_{2/8}},$$

where the quantities $Q_{7/8},Q_{5/8},Q_{1/8}$, and $Q_{3/8}$ in Eq. (7) can be established by employing $p=\frac{7}{8},\frac{5}{8},\frac{1}{8}$, and $p=\frac{3}{8}$ in Eq. (5), respectively.

### 3.2 The $r^{th}$ moment

For the NFWE distribution with PDF in Eq. (4), the $r^{th}$ moment is

$$\mu_{r}^{/}=\int_{0}^{\infty }u^{r}\frac{\left(\delta_{1}+\frac{\delta_{2}}{u^{2}}\right)e^{\delta_{1}u-\delta_{2}/u}e^{-e^{\delta_{1}u-\delta_{2}/u}}}{\exp\left\{1-e^{-e^{\delta_{1}u-\delta_{2}/u}}\right\}}\left[1+e^{-e^{\delta_{1}u-\delta_{2}/u}}\right]du.$$

On solving, we observe

$$\mu_{r}^{/}=\sum_{i=0}^{1}\sum_{j,k,l,m}^{\infty }\left(\begin{array}{c} 1\\ i \end{array}\right)\frac{{\left(-1\right)}^{j+k+l+m}{\left(k+1\right)}^{l}{\left(\delta_{2}l\right)}^{m}}{i!m!2^{i-1}}\int_{0}^{\infty }u^{r}\left(\delta_{1}+\frac{\delta_{2}}{u^{2}}\right)e^{m\delta_{1}u}du,$$

$$\mu_{r}^{/}=\sum_{i=0}^{1}\sum_{j,k,l,m}^{\infty }\left(\begin{array}{c} 1\\ i \end{array}\right)\frac{{\left(-1\right)}^{j+k+l+m}{\left(k+1\right)}^{l}{\left(\delta_{2}l\right)}^{m}}{i!m!2^{i-1}}\left(\delta_{1}\int_{0}^{\infty }u^{r}e^{m\delta_{1}u}du+\delta_{2}\int_{0}^{\infty }u^{r-2}e^{m\delta_{1}u}du\right),$$

$$\mu_{r}^{/}=\sum_{i=0}^{1}\sum_{j,k,l,m}^{\infty }\left(\begin{array}{c} 1\\ i \end{array}\right)\frac{{\left(-1\right)}^{j+k+l+m}{\left(k+1\right)}^{l}{\left(\delta_{2}l\right)}^{m}}{i!m!2^{i-1}}\left[\delta_{1}\kappa_{1}\left(u\right)+\delta_{2}\kappa_{2}\left(u\right)\right],$$

where

$$\kappa_{1}\left(u\right)=\int_{0}^{\infty }u^{r}e^{m\delta_{1}u}du,$$

and

$$\kappa_{2}\left(u\right)=\int_{0}^{\infty }u^{r-2}e^{m\delta_{1}u}du.$$

## 4 Parameters estimation

This section explores the frequently implemented estimation approaches to obtain the estimators $\left({\overset{ˆ}{\delta }}_{1},{\overset{ˆ}{\delta }}_{2}\right)$ of the parameters $\left(\delta_{1},\delta_{2}\right)$ of the NFWE distribution. These estimators include the weighted least-square (WLS) estimators, ordinary least-square (OLS) estimators, maximum likelihood (ML) estimators, maximum product spacing (MPS) estimators, Cramér–von Mises (CVM) estimators, Anderson–Darling (AD) estimators, Right-tail Anderson–Darling (RAD) estimators and percentile (PC) estimators. Recently, these methods have also been applied by [20], [21] for estimating the model parameters.

### 4.1 The ML estimators

Consider a random sample $U_{1},U_{2},...,U_{n}$ from g(u;Ψ). Corresponding to g(u;Ψ), the log-likelihood function $\ell \left(\delta_{1},\delta_{2}\right)$ is

$$\ell \left(\delta_{1},\delta_{2}\right)=\sum_{r=i}^{n}\log\left(\delta_{1}+\frac{\delta_{2}}{u_{r}^{2}}\right)+\sum_{r=i}^{n}\left(\delta_{1}u_{r}-\frac{\delta_{2}}{u_{r}}\right)-\sum_{r=i}^{n}e^{\delta_{1}u_{r}-\frac{\delta_{2}}{u_{r}^{2}}}+\sum_{r=i}^{n}\log\left(1+e^{-e^{\delta_{1}u_{r}-\frac{\delta_{2}}{u_{r}}}}\right)-\sum_{r=i}^{n}\left\{1-e^{-e^{\delta_{1}u_{r}-\frac{\delta_{2}}{u_{r}}}}\right\}.$$

The partial derivatives of $\ell \left(\delta_{1},\delta_{2}\right)$ on behalf of $\delta_{1}$ and $\delta_{2}$ are given, respectively, by

$$\frac{\partial }{\partial \delta_{1}}\ell \left(\delta_{1},\delta_{2}\right)=\sum_{r=i}^{n}\frac{1}{\left(\delta_{1}+\frac{\delta_{2}}{u_{r}^{2}}\right)}+\sum_{r=i}^{n}u_{r}-\sum_{r=i}^{n}u_{r}e^{\delta_{1}u_{r}-\frac{\delta_{2}}{u_{r}^{2}}}-\sum_{r=i}^{n}\frac{u_{r}e^{\delta_{1}u_{r}-\frac{\delta_{2}}{u_{r}^{2}}}e^{-e^{\delta_{1}u_{r}-\frac{\delta_{2}}{u_{r}}}}}{\left(1+e^{-e^{\delta_{1}u_{r}-\frac{\delta_{2}}{u_{r}}}}\right)}-\sum_{r=i}^{n}u_{r}e^{\delta_{1}u_{r}-\frac{\delta_{2}}{u_{r}^{2}}}e^{-e^{\delta_{1}u_{r}-\frac{\delta_{2}}{u_{r}}}},$$

and

$$\frac{\partial }{\partial \delta_{2}}\ell \left(\delta_{1},\delta_{2}\right)=\sum_{r=i}^{n}\frac{\frac{1}{u_{r}^{2}}}{\left(\delta_{1}+\frac{\delta_{2}}{u_{r}^{2}}\right)}-\sum_{r=i}^{n}\frac{1}{u_{r}}+\sum_{r=i}^{n}\frac{1}{u_{r}}e^{\delta_{1}u_{r}-\frac{\delta_{2}}{u_{r}^{2}}}+\sum_{r=i}^{n}\frac{\frac{1}{u_{r}}e^{\delta_{1}u_{r}-\frac{\delta_{2}}{u_{r}^{2}}}e^{-e^{\delta_{1}u_{r}-\frac{\delta_{2}}{u_{r}}}}}{\left(1+e^{-e^{\delta_{1}u_{r}-\frac{\delta_{2}}{u_{r}}}}\right)}+\sum_{r=i}^{n}\frac{1}{u_{r}}e^{\delta_{1}u_{r}-\frac{\delta_{2}}{u_{r}^{2}}}e^{-e^{\delta_{1}u_{r}-\frac{\delta_{2}}{u_{r}}}}.$$

On solving $\frac{\partial }{\partial \delta_{1}}\ell (\delta_{1},$ $\delta_{2})=0$ and $\frac{\partial }{\partial \delta_{2}}\ell \left(\delta_{1},\delta_{2}\right)=0$, we obtain the MLEs $\left(\overset{ˆ}{\delta_{1}},\overset{ˆ}{\delta_{2}}\right)$ of $\left(\delta_{1},\delta_{2}\right)$.

### 4.2 The OLS and WLS estimators

Let $u_{\left(1\right)},u_{\left(2\right)},\cdots ,u_{\left(n\right)}$ be a set of the order statistics selected from $G\left(\text{u};\delta_{1},\delta_{2}\right)$ in Eq. (3). The OLS estimators (see [22]) ${\overset{ˆ}{\delta_{1}}}_{LSE}$ and ${\overset{ˆ}{\delta_{2}}}_{LSE}$ can be attained through minimizing

$$V\left(\delta_{1},\delta_{2}\right)=\sum_{i=1}^{n}{\left[G\left(u_{\left(i\right)}|\delta_{1},\delta_{2}\right)-\frac{i}{n+1}\right]}^{2},$$

with respect to $\delta_{1}$ and $\delta_{2}$.

We can also attain the OLS estimators ${\overset{ˆ}{\delta_{1}}}_{LSE}$ and ${\overset{ˆ}{\delta_{2}}}_{LSE}$, by solving

$$\sum_{i=1}^{n}\left[G\left(u_{\left(i\right)}|\delta_{1},\delta_{2}\right)-\frac{i}{n+1}\right]\Delta_{s}\left(u_{\left(i\right)}|\delta_{1},\delta_{2}\right)=0,\;\;s=1,2,$$

where

(8) $$\Delta_{1}\left(u_{\left(i\right)}|\delta_{1},\delta_{2}\right)=\frac{\partial }{\partial \delta_{1}}G\left(u_{\left(i\right)}|\delta_{1},\delta_{2}\right),$$

and

(9) $$\Delta_{2}\left(u_{\left(i\right)}|\delta_{1},\delta_{2}\right)=\frac{\partial }{\partial \delta_{2}}G\left(u_{\left(i\right)}|\delta_{1},\delta_{2}\right).$$

Furthermore, we attain the WLS estimators ${\overset{ˆ}{\delta_{1}}}_{WLSE}$ and ${\overset{ˆ}{\delta_{2}}}_{WLSE}$, by solving

$$\sum_{i=1}^{n}\frac{{\left(n+1\right)}^{2}\left(n+2\right)}{i\left(n-i+1\right)}\left[G\left(u_{\left(i\right)}|\delta_{1},\delta_{2}\right)-\frac{i}{n+1}\right]\Delta_{s}\left(u_{\left(i\right)}|\delta_{1},\delta_{2}\right)=0,$$

where the quantities $\Delta_{1}\left(\cdot |\delta_{1},\delta_{2}\right)$ and $\Delta_{2}\left(\cdot |\delta_{1},\delta_{2}\right)$ are given in Eq. (8) and Eq. (9), respectively.

### 4.3 The MPS estimators

The MPS estimators ([23], [24]), say ${\overset{ˆ}{\delta_{1}}}_{MPSE}$ and ${\overset{ˆ}{\delta_{2}}}_{MPSE}$, of the NFWE distribution are attained by solving

$$\frac{1}{n+1}\sum_{i=1}^{n+1}\frac{1}{D_{i}(\delta_{1},\delta_{2})}\left[\Delta_{s}(u_{\left(i\right)}|\delta_{1},\delta_{2})-\Delta_{s}(u_{\left(i-1\right)}|\delta_{1},\delta_{2})\right]=0,$$

where the quantities $\Delta_{1}\left(\cdot |\delta_{1},\delta_{2}\right)$ and $\Delta_{2}\left(\cdot |\delta_{1},\delta_{2}\right)$ are given in Eq. (8) and Eq. (9), respectively.

### 4.4 The CVM estimators

The CVM estimators [25], say ${\overset{ˆ}{\delta_{1}}}_{MPSE}$ and ${\overset{ˆ}{\delta_{2}}}_{MPSE}$, of the NFWE distribution are attained by solving

$$\sum_{i=1}^{n}\left[G\left(u_{\left(i\right)}|\delta_{1},\delta_{2}\right)-\frac{2i-1}{2n}\right]\Delta_{s}\left(u_{\left(i\right)}|\delta_{1},\delta_{2}\right)=0,$$

where the quantities $\Delta_{1}\left(\cdot |\delta_{1},\delta_{2}\right)$ and $\Delta_{2}\left(\cdot |\delta_{1},\delta_{2}\right)$ are given in Eq. (8) and Eq. (9), respectively.

### 4.5 The AD and RAD estimators

The AD estimators [26], say ${\overset{ˆ}{\delta_{1}}}_{MPSE}$ and ${\overset{ˆ}{\delta_{2}}}_{MPSE}$, of the NFWE distribution are attained by solving

$$A(\delta_{1},\delta_{2})=-n-\frac{1}{n}\sum_{i=1}^{n}\left(2i-1\right)\;\left[\log G\left(u_{\left(i\right)}|\delta_{1},\delta_{2}\right)+\log S\left(u_{\left(i\right)}|\delta_{1},\delta_{2}\right)\right],$$

with respected to $\delta_{1}$ and $\delta_{2}$.

For the NFWE distribution, the RAD estimators, say ${\overset{ˆ}{\delta_{1}}}_{MPSE}$ and ${\overset{ˆ}{\delta_{2}}}_{MPSE}$, are attained by solving

$$R(\delta_{1},\delta_{2})=\frac{n}{2}-2\sum_{i=1}^{n}G\left(u_{i:n}|\delta_{1},\delta_{2}\right)-\frac{1}{n}\sum_{i=1}^{n}\left(2i-1\right)\log S\left(u_{n+1-i:n}|\delta_{1},\delta_{2}\right).$$

### 4.6 The PC estimators

The percentile estimation method is another most prominent method for estimating the papers [27], [28]. For the NFWE distribution, the PC estimators, say ${\overset{ˆ}{\delta_{1}}}_{MPSE}$ and ${\overset{ˆ}{\delta_{2}}}_{MPSE}$, are attained by minimizing

$$P(\delta_{1},\delta_{2})=\sum_{i=1}^{n}{\left(u_{\left(i\right)}-Q(u_{i})\right)}^{2},$$

where Q(⋅) is the QF.

## 5 Simulation study

This section demonstrates the performances of $\overset{ˆ}{\delta_{1}}$ and $\overset{ˆ}{\delta_{2}}$ of the NFWE distribution by employing the MC (Monte Carlo) simulation study. The simulation results are obtained for thirty-six distinct sets of parameters when: $\delta_{1}=\{0.10,0.50,0.95$, 1.56,2.21,3.00} and $\delta_{2}=\{0.05,0.45,0.87,1.39,2.15,4.00\}$.

For demonstrating the performances of $\overset{ˆ}{\delta_{1}}$ and $\overset{ˆ}{\delta_{2}}$, we attain different random samples: $U_{1},U_{2},\ldots ,U_{N}$ of sizes n=20,50,80,120,200 and 300 from the NFWE distribution by using Eq. (5) and simulate them N=5000 times. The simulation is done by utilizing the R statistical software (version 4.1.1) with nlminb() function. For demonstrating the performances of $\overset{ˆ}{\delta_{1}}$ and $\overset{ˆ}{\delta_{2}}$, take into account four judgment tools (also called statistical criteria). These criteria include

• Absolute biases, which is computed as
  $$|Bias(\overset{ˆ}{\Psi })|=\frac{1}{5000}\sum_{i=1}^{5000}|\overset{ˆ}{\Psi }-\Psi |,$$
• Mean square errors (MSEs), which is calculated as
  $$MSEs(\overset{ˆ}{\Psi })=\frac{1}{5000}\sum_{i=1}^{5000}{\left(\overset{ˆ}{\Psi }-\Psi \right)}^{2},$$
  and
• Mean relative errors (MREs), which is obtained as
  $$MREs(\overset{ˆ}{\Psi })=\frac{1}{5000}\sum_{i=1}^{5000}|\overset{ˆ}{\Psi }-\Psi |/\Psi .$$

Out of many simulated outcomes (thirty-six outcomes), we only report the simulation results for six combination values of $\delta_{1}=\{0.10,0.50,0.95$, 1.56,2.21,3.00} and $\delta_{2}=\{0.05,0.45,0.87,1.39,2.15,4.00\}$. For the given values of $\delta_{1}$ and $\delta_{1}$, Table 1, Table 2, Table 3, Table 4, Table 5, Table 6 provide the simulation results of the NFWE distribution. Table 1, Table 2, Table 3, Table 4, Table 5, Table 6 also provide the rank of the estimators presented in the superscript indicators. The ∑Ranks is also provided in Table 1, Table 2, Table 3, Table 4, Table 5, Table 6. In addition to Table 1, Table 2, Table 3, Table 4, Table 5, Table 6, Table 7 summarizes the partial as well as the overall ranks of $\delta_{1}$ and $\delta_{1}$. Corresponding to the given facts in Table 1, Table 2, Table 3, Table 4, Table 5, Table 6, Table 7, we can easily pick the following conclusion:

**Table 1:** Simulation results for $\Psi ={\left(\delta_{1}=0.10,\delta_{2}=0.05\right)}^{⊺}$.

*n*	Est.	Est. Par.	WLS	OLS	ML	MPS	CVM	AD	RAD	PC
20	|*BIAS*|	${\overset{ˆ}{\delta }}_{1}$	0.24280^{6}^	0.27451^{7}^	0.09078^{3}^	0.06287^{1}^	0.46514^{8}^	0.10272^{5}^	0.09215^{4}^	0.06993^{2}^
		${\overset{ˆ}{\delta }}_{2}$	0.01972^{4}^	0.02159^{5}^	0.01662^{2}^	0.01633^{1}^	0.02172^{6}^	0.01890^{3}^	0.03003^{7}^	0.26821^{8}^
	*MSE*	${\overset{ˆ}{\delta }}_{1}$	0.77797^{6}^	0.88629^{7}^	0.10085^{4}^	0.07632^{2}^	1.78528^{8}^	0.13545^{5}^	0.09673^{3}^	0.05492^{1}^
		${\overset{ˆ}{\delta }}_{2}$	0.00100^{4}^	0.00168^{6}^	0.00048^{2}^	0.00042^{1}^	0.00125^{5}^	0.00068^{3}^	0.01642^{7}^	0.19259^{8}^
	*MRE*	${\overset{ˆ}{\delta }}_{1}$	2.42799^{6}^	2.74513^{7}^	0.90782^{3}^	0.62866^{1}^	4.65144^{8}^	1.02720^{5}^	0.92149^{4}^	0.69928^{2}^
		${\overset{ˆ}{\delta }}_{2}$	0.19716^{4}^	0.21589^{5}^	0.16616^{2}^	0.16330^{1}^	0.21722^{6}^	0.18900^{3}^	0.30035^{7}^	2.68212^{8}^
	∑*Ranks*		30^{5}^	37^{7}^	16^{2}^	7^{1}^	41^{8}^	24^{3}^	32^{6}^	29^{4}^

50	|*BIAS*|	${\overset{ˆ}{\delta }}_{1}$	0.07789^{6}^	0.11256^{7}^	0.02376^{2}^	0.02231^{1}^	0.15062^{8}^	0.03080^{5}^	0.02991^{4}^	0.02385^{3}^
		${\overset{ˆ}{\delta }}_{2}$	0.01160^{3}^	0.01243^{5}^	0.01012^{2}^	0.00996^{1}^	0.01280^{6}^	0.01171^{4}^	0.01426^{7}^	0.15050^{8}^
	*MSE*	${\overset{ˆ}{\delta }}_{1}$	0.09689^{6}^	0.13843^{7}^	0.00140^{3}^	0.00098^{1}^	0.19853^{8}^	0.00243^{5}^	0.00238^{4}^	0.00130^{2}^
		${\overset{ˆ}{\delta }}_{2}$	0.00023^{3.5}^	0.00026^{5}^	0.00017^{2}^	0.00015^{1}^	0.00029^{6}^	0.00023^{3.5}^	0.00037^{7}^	0.05272^{8}^
	*MRE*	${\overset{ˆ}{\delta }}_{1}$	0.77890^{6}^	1.12556^{7}^	0.23760^{2}^	0.22306^{1}^	1.50619^{8}^	0.30800^{5}^	0.29915^{4}^	0.23853^{3}^
		${\overset{ˆ}{\delta }}_{2}$	0.11604^{3}^	0.12432^{5}^	0.10123^{2}^	0.09956^{1}^	0.12797^{6}^	0.11714^{4}^	0.14265^{7}^	1.50496^{8}^
	∑*Ranks*		27.5^{4}^	36^{7}^	13^{2}^	6^{1}^	42^{8}^	26.5^{3}^	33^{6}^	32^{5}^

80	|*BIAS*|	${\overset{ˆ}{\delta }}_{1}$	0.04017^{6}^	0.07155^{7}^	0.01709^{2}^	0.01619^{1}^	0.08665^{8}^	0.02315^{5}^	0.02204^{4}^	0.01743^{3}^
		${\overset{ˆ}{\delta }}_{2}$	0.00927^{4}^	0.00981^{6}^	0.00777^{1}^	0.00796^{2}^	0.00960^{5}^	0.00910^{3}^	0.01130^{7}^	0.11841^{8}^
	*MSE*	${\overset{ˆ}{\delta }}_{1}$	0.01803^{6}^	0.05238^{7}^	0.00060^{3}^	0.00043^{1}^	0.07471^{8}^	0.00111^{5}^	0.00096^{4}^	0.00056^{2}^
		${\overset{ˆ}{\delta }}_{2}$	0.00014^{4}^	0.00016^{5.5}^	0.00010^{1.5}^	0.00010^{1.5}^	0.00016^{5.5}^	0.00013^{3}^	0.00022^{7}^	0.02964^{8}^
	*MRE*	${\overset{ˆ}{\delta }}_{1}$	0.40167^{6}^	0.71547^{7}^	0.17093^{2}^	0.16188^{1}^	0.86652^{8}^	0.23151^{5}^	0.22041^{4}^	0.17431^{3}^
		${\overset{ˆ}{\delta }}_{2}$	0.09269^{4}^	0.09814^{6}^	0.07769^{1}^	0.07955^{2}^	0.09596^{5}^	0.09102^{3}^	0.11297^{7}^	1.18413^{8}^
	∑*Ranks*		30^{4}^	38.5^{7}^	10.5^{2}^	8.5^{1}^	39.5^{8}^	24^{3}^	33^{6}^	32^{5}^

120	|*BIAS*|	${\overset{ˆ}{\delta }}_{1}$	0.02750^{6}^	0.04292^{7}^	0.01299^{2}^	0.01277^{1}^	0.05008^{8}^	0.01829^{5}^	0.01764^{4}^	0.01350^{3}^
		${\overset{ˆ}{\delta }}_{2}$	0.00745^{4}^	0.00808^{5.5}^	0.00636^{1}^	0.00638^{2}^	0.00808^{5.5}^	0.00723^{3}^	0.00907^{7}^	0.09546^{8}^
	*MSE*	${\overset{ˆ}{\delta }}_{1}$	0.00807^{6}^	0.01470^{7}^	0.00031^{3}^	0.00026^{1}^	0.01906^{8}^	0.00061^{5}^	0.00055^{4}^	0.00030^{2}^
		${\overset{ˆ}{\delta }}_{2}$	0.00009^{4}^	0.00011^{5.5}^	0.00007^{2}^	0.00006^{1}^	0.00011^{5.5}^	0.00008^{3}^	0.00014^{7}^	0.01778^{8}^
	*MRE*	${\overset{ˆ}{\delta }}_{1}$	0.27502^{6}^	0.42919^{7}^	0.12987^{2}^	0.12766^{1}^	0.50084^{8}^	0.18294^{5}^	0.17637^{4}^	0.13501^{3}^
		${\overset{ˆ}{\delta }}_{2}$	0.07447^{4}^	0.08084^{5}^	0.06361^{1}^	0.06377^{2}^	0.08085^{6}^	0.07233^{3}^	0.09066^{7}^	0.95461^{8}^
	∑*Ranks*		30^{4}^	37^{7}^	11^{2}^	8^{1}^	41^{8}^	24^{3}^	33^{6}^	32^{5}^

200	|*BIAS*|	${\overset{ˆ}{\delta }}_{1}$	0.01650^{6}^	0.02581^{7}^	0.00962^{2}^	0.00949^{1}^	0.02784^{8}^	0.01365^{5}^	0.01327^{4}^	0.00995^{3}^
		${\overset{ˆ}{\delta }}_{2}$	0.00565^{4}^	0.00622^{6}^	0.00492^{1}^	0.00493^{2}^	0.00613^{5}^	0.00564^{3}^	0.00698^{7}^	0.07420^{8}^
	*MSE*	${\overset{ˆ}{\delta }}_{1}$	0.00124^{6}^	0.00262^{7}^	0.00016^{2.5}^	0.00014^{1}^	0.00459^{8}^	0.00032^{5}^	0.00030^{4}^	0.00016^{2.5}^
		${\overset{ˆ}{\delta }}_{2}$	0.00005^{3.5}^	0.00006^{5.5}^	0.00004^{1.5}^	0.00004^{1.5}^	0.00006^{5.5}^	0.00005^{3.5}^	0.00008^{7}^	0.01010^{8}^
	*MRE*	${\overset{ˆ}{\delta }}_{1}$	0.16501^{6}^	0.25815^{7}^	0.09622^{2}^	0.09492^{1}^	0.27839^{8}^	0.13649^{5}^	0.13273^{4}^	0.09948^{3}^
		${\overset{ˆ}{\delta }}_{2}$	0.05646^{4}^	0.06216^{6}^	0.04915^{1}^	0.04931^{2}^	0.06128^{5}^	0.05638^{3}^	0.06975^{7}^	0.74200^{8}^
	∑*Ranks*		29.5^{4}^	38.5^{7}^	10^{2}^	8.5^{1}^	39.5^{8}^	24.5^{3}^	33^{6}^	32.5^{5}^

300	|*BIAS*|	${\overset{ˆ}{\delta }}_{1}$	0.01197^{6}^	0.01885^{7}^	0.00763^{1}^	0.00775^{2}^	0.01959^{8}^	0.01123^{5}^	0.01070^{4}^	0.00812^{3}^
		${\overset{ˆ}{\delta }}_{2}$	0.00463^{4}^	0.00508^{5.5}^	0.00398^{2}^	0.00395^{1}^	0.00508^{5.5}^	0.00462^{3}^	0.00568^{7}^	0.05980^{8}^
	*MSE*	${\overset{ˆ}{\delta }}_{1}$	0.00027^{6}^	0.00099^{7}^	0.00010^{2}^	0.00009^{1}^	0.00102^{8}^	0.00021^{5}^	0.00019^{4}^	0.00011^{3}^
		${\overset{ˆ}{\delta }}_{2}$	0.00003^{3}^	0.00004^{5.5}^	0.00003^{3}^	0.00002^{1}^	0.00004^{5.5}^	0.00003^{3}^	0.00005^{7}^	0.00630^{8}^
	*MRE*	${\overset{ˆ}{\delta }}_{1}$	0.11969^{6}^	0.18850^{7}^	0.07630^{1}^	0.07755^{2}^	0.19587^{8}^	0.11228^{5}^	0.10696^{4}^	0.08123^{3}^
		${\overset{ˆ}{\delta }}_{2}$	0.04628^{4}^	0.05080^{5.5}^	0.03984^{2}^	0.03947^{1}^	0.05080^{5.5}^	0.04625^{3}^	0.05681^{7}^	0.59800^{8}^
	∑*Ranks*		29^{4}^	37.5^{7}^	11^{2}^	8^{1}^	40.5^{8}^	24^{3}^	33^{5.5}^	33^{5.5}^

**Table 2:** Simulation results for $\Psi ={\left(\delta_{1}=0.50,\delta_{2}=0.05\right)}^{⊺}$.

*n*	Est.	Est. Par.	WLS	OLS	ML	MPS	CVM	AD	RAD	PC
20	|*BIAS*|	${\overset{ˆ}{\delta }}_{1}$	0.47541^{6}^	0.57863^{7}^	0.30634^{5}^	0.22250^{1}^	0.76841^{8}^	0.30284^{4}^	0.29289^{3}^	0.23438^{2}^
		${\overset{ˆ}{\delta }}_{2}$	0.01827^{3.5}^	0.01968^{5}^	0.01664^{2}^	0.01628^{1}^	0.02181^{6}^	0.01827^{3.5}^	0.02431^{7}^	0.07411^{8}^
	*MSE*	${\overset{ˆ}{\delta }}_{1}$	1.28588^{6}^	1.59033^{7}^	0.38167^{5}^	0.16758^{1}^	2.81926^{8}^	0.33148^{3}^	0.37551^{4}^	0.23544^{2}^
		${\overset{ˆ}{\delta }}_{2}$	0.00062^{4}^	0.00081^{5}^	0.00049^{2}^	0.00042^{1}^	0.00106^{6}^	0.00059^{3}^	0.00137^{7}^	0.01128^{8}^
	*MRE*	${\overset{ˆ}{\delta }}_{1}$	0.95082^{6}^	1.15726^{7}^	0.61268^{5}^	0.44500^{1}^	1.53682^{8}^	0.60568^{4}^	0.58577^{3}^	0.46877^{2}^
		${\overset{ˆ}{\delta }}_{2}$	0.18274^{4}^	0.19677^{5}^	0.16636^{2}^	0.16275^{1}^	0.21806^{6}^	0.18268^{3}^	0.24309^{7}^	0.74107^{8}^
	∑*Ranks*		29.5^{4}^	36^{7}^	21^{3}^	6^{1}^	42^{8}^	20.5^{2}^	31^{6}^	30^{5}^

50	|*BIAS*|	${\overset{ˆ}{\delta }}_{1}$	0.23679^{6}^	0.29489^{7}^	0.11716^{3}^	0.10810^{2}^	0.32363^{8}^	0.14863^{5}^	0.13704^{4}^	0.10627^{1}^
		${\overset{ˆ}{\delta }}_{2}$	0.01102^{3}^	0.01182^{5}^	0.01006^{2}^	0.01004^{1}^	0.01221^{6}^	0.01126^{4}^	0.01398^{7}^	0.04864^{8}^
	*MSE*	${\overset{ˆ}{\delta }}_{1}$	0.24478^{6}^	0.32416^{7}^	0.03121^{3}^	0.02211^{1}^	0.38260^{8}^	0.04779^{5}^	0.03935^{4}^	0.02238^{2}^
		${\overset{ˆ}{\delta }}_{2}$	0.00020^{3}^	0.00024^{5}^	0.00017^{2}^	0.00016^{1}^	0.00026^{6}^	0.00021^{4}^	0.00034^{7}^	0.00423^{8}^
	*MRE*	${\overset{ˆ}{\delta }}_{1}$	0.47357^{6}^	0.58979^{7}^	0.23432^{3}^	0.21620^{2}^	0.64725^{8}^	0.29726^{5}^	0.27407^{4}^	0.21254^{1}^
		${\overset{ˆ}{\delta }}_{2}$	0.11020^{3}^	0.11822^{5}^	0.10064^{2}^	0.10040^{1}^	0.12211^{6}^	0.11258^{4}^	0.13980^{7}^	0.48644^{8}^
	∑*Ranks*		27^{3.5}^	36^{7}^	15^{2}^	8^{1}^	42^{8}^	27^{3.5}^	33^{6}^	28^{5}^

80	|*BIAS*|	${\overset{ˆ}{\delta }}_{1}$	0.14931^{6}^	0.20723^{7}^	0.08219^{3}^	0.08008^{2}^	0.21985^{8}^	0.11415^{5}^	0.10372^{4}^	0.08006^{1}^
		${\overset{ˆ}{\delta }}_{2}$	0.00897^{4}^	0.00928^{5}^	0.00788^{1}^	0.00792^{2}^	0.00946^{6}^	0.00884^{3}^	0.01081^{7}^	0.03737^{8}^
	*MSE*	${\overset{ˆ}{\delta }}_{1}$	0.06964^{6}^	0.12987^{7}^	0.01372^{3}^	0.01054^{1}^	0.14508^{8}^	0.02401^{5}^	0.02062^{4}^	0.01115^{2}^
		${\overset{ˆ}{\delta }}_{2}$	0.00013^{3.5}^	0.00014^{5}^	0.00010^{1.5}^	0.00010^{1.5}^	0.00015^{6}^	0.00013^{3.5}^	0.00020^{7}^	0.00236^{8}^
	*MRE*	${\overset{ˆ}{\delta }}_{1}$	0.29862^{6}^	0.41446^{7}^	0.16437^{3}^	0.16016^{2}^	0.43970^{8}^	0.22830^{5}^	0.20745^{4}^	0.16012^{1}^
		${\overset{ˆ}{\delta }}_{2}$	0.08965^{4}^	0.09285^{5}^	0.07877^{1}^	0.07915^{2}^	0.09465^{6}^	0.08836^{3}^	0.10809^{7}^	0.37371^{8}^
	∑*Ranks*		29.5^{5}^	36^{7}^	12.5^{2}^	10.5^{1}^	42^{8}^	24.5^{3}^	33^{6}^	28^{4}^

120	|*BIAS*|	${\overset{ˆ}{\delta }}_{1}$	0.10981^{6}^	0.15282^{7}^	0.06530^{3}^	0.06338^{2}^	0.16171^{8}^	0.08988^{5}^	0.08205^{4}^	0.06292^{1}^
		${\overset{ˆ}{\delta }}_{2}$	0.00707^{3}^	0.00758^{5}^	0.00644^{2}^	0.00637^{1}^	0.00772^{6}^	0.00716^{4}^	0.00871^{7}^	0.03109^{8}^
	*MSE*	${\overset{ˆ}{\delta }}_{1}$	0.03241^{6}^	0.05512^{7}^	0.00775^{3}^	0.00632^{1}^	0.06526^{8}^	0.01409^{5}^	0.01172^{4}^	0.00653^{2}^
		${\overset{ˆ}{\delta }}_{2}$	0.00008^{3.5}^	0.00009^{5}^	0.00007^{2}^	0.00006^{1}^	0.00010^{6}^	0.00008^{3.5}^	0.00012^{7}^	0.00158^{8}^
	*MRE*	${\overset{ˆ}{\delta }}_{1}$	0.21961^{6}^	0.30565^{7}^	0.13060^{3}^	0.12675^{2}^	0.32342^{8}^	0.17976^{5}^	0.16410^{4}^	0.12585^{1}^
		${\overset{ˆ}{\delta }}_{2}$	0.07070^{3}^	0.07581^{5}^	0.06436^{2}^	0.06374^{1}^	0.07719^{6}^	0.07162^{4}^	0.08706^{7}^	0.31085^{8}^
	∑*Ranks*		27.5^{4}^	36^{7}^	15^{2}^	8^{1}^	42^{8}^	26.5^{3}^	33^{6}^	28^{5}^

200	|*BIAS*|	${\overset{ˆ}{\delta }}_{1}$	0.07460^{6}^	0.11070^{7}^	0.04740^{1}^	0.04871^{2}^	0.11559^{8}^	0.06960^{5}^	0.06319^{4}^	0.04881^{3}^
		${\overset{ˆ}{\delta }}_{2}$	0.00547^{3}^	0.00583^{5}^	0.00497^{1}^	0.00502^{2}^	0.00588^{6}^	0.00564^{4}^	0.00669^{7}^	0.02411^{8}^
	*MSE*	${\overset{ˆ}{\delta }}_{1}$	0.01116^{6}^	0.02437^{7}^	0.00388^{3}^	0.00369^{1}^	0.02819^{8}^	0.00829^{5}^	0.00668^{4}^	0.00371^{2}^
		${\overset{ˆ}{\delta }}_{2}$	0.00005^{4}^	0.00005^{4}^	0.00004^{1.5}^	0.00004^{1.5}^	0.00006^{6}^	0.00005^{4}^	0.00007^{7}^	0.00094^{8}^
	*MRE*	${\overset{ˆ}{\delta }}_{1}$	0.14921^{6}^	0.22139^{7}^	0.09479^{1}^	0.09743^{2}^	0.23118^{8}^	0.13920^{5}^	0.12638^{4}^	0.09762^{3}^
		${\overset{ˆ}{\delta }}_{2}$	0.05467^{3}^	0.05832^{5}^	0.04970^{1}^	0.05018^{2}^	0.05877^{6}^	0.05644^{4}^	0.06695^{7}^	0.24107^{8}^
	∑*Ranks*		28^{4}^	35^{7}^	8.5^{1}^	10.5^{2}^	42^{8}^	27^{3}^	33^{6}^	32^{5}^

300	|*BIAS*|	${\overset{ˆ}{\delta }}_{1}$	0.05805^{6}^	0.08724^{7}^	0.03798^{1}^	0.03864^{2}^	0.08848^{8}^	0.05644^{5}^	0.05147^{4}^	0.03908^{3}^
		${\overset{ˆ}{\delta }}_{2}$	0.00449^{4}^	0.00475^{5}^	0.00406^{1}^	0.00412^{2}^	0.00490^{6}^	0.00448^{3}^	0.00540^{7}^	0.02019^{8}^
	*MSE*	${\overset{ˆ}{\delta }}_{1}$	0.00603^{6}^	0.01399^{7}^	0.00239^{2}^	0.00229^{1}^	0.01503^{8}^	0.00522^{5}^	0.00444^{4}^	0.00243^{3}^
		${\overset{ˆ}{\delta }}_{2}$	0.00003^{2.5}^	0.00004^{5.5}^	0.00003^{2.5}^	0.00003^{2.5}^	0.00004^{5.5}^	0.00003^{2.5}^	0.00005^{7}^	0.00064^{8}^
	*MRE*	${\overset{ˆ}{\delta }}_{1}$	0.11610^{6}^	0.17448^{7}^	0.07596^{1}^	0.07727^{2}^	0.17697^{8}^	0.11288^{5}^	0.10294^{4}^	0.07817^{3}^
		${\overset{ˆ}{\delta }}_{2}$	0.04494^{4}^	0.04746^{5}^	0.04060^{1}^	0.04122^{2}^	0.04900^{6}^	0.04484^{3}^	0.05404^{7}^	0.20191^{8}^
	∑*Ranks*		28.5^{4}^	36.5^{7}^	8.5^{1}^	11.5^{2}^	41.5^{8}^	23.5^{3}^	33^{5.5}^	33^{5.5}^

**Table 3:** Simulation results for $\Psi ={\left(\delta_{1}=0.95,\delta_{2}=0.45\right)}^{⊺}$.

*n*	Est.	Est. Par.	WLS	OLS	ML	MPS	CVM	AD	RAD	PC
20	|*BIAS*|	${\overset{ˆ}{\delta }}_{1}$	0.32922^{6}^	0.36144^{7}^	0.28780^{5}^	0.25174^{1}^	0.42559^{8}^	0.28500^{4}^	0.28042^{3}^	0.25997^{2}^
		${\overset{ˆ}{\delta }}_{2}$	0.08086^{3}^	0.08425^{5}^	0.08127^{4}^	0.07618^{1}^	0.09290^{6}^	0.08019^{2}^	0.09449^{7}^	0.10218^{8}^
	*MSE*	${\overset{ˆ}{\delta }}_{1}$	0.25719^{6}^	0.27306^{7}^	0.18212^{5}^	0.10387^{1}^	0.43870^{8}^	0.15397^{3}^	0.15963^{4}^	0.11739^{2}^
		${\overset{ˆ}{\delta }}_{2}$	0.01129^{3}^	0.01226^{5}^	0.01150^{4}^	0.00911^{1}^	0.01607^{7}^	0.01122^{2}^	0.01604^{6}^	0.01692^{8}^
	*MRE*	${\overset{ˆ}{\delta }}_{1}$	0.34655^{6}^	0.38046^{7}^	0.30295^{5}^	0.26499^{1}^	0.44799^{8}^	0.30000^{4}^	0.29518^{3}^	0.27366^{2}^
		${\overset{ˆ}{\delta }}_{2}$	0.16173^{3}^	0.16850^{5}^	0.16255^{4}^	0.15237^{1}^	0.18579^{6}^	0.16038^{2}^	0.18898^{7}^	0.20436^{8}^
	∑*Ranks*		27^{3.5}^	36^{7}^	27^{3.5}^	6^{1}^	43^{8}^	17^{2}^	30^{5.5}^	30^{5.5}^

50	|*BIAS*|	${\overset{ˆ}{\delta }}_{1}$	0.18658^{6}^	0.21618^{7}^	0.15155^{2}^	0.14836^{1}^	0.22918^{8}^	0.17008^{5}^	0.16266^{4}^	0.15314^{3}^
		${\overset{ˆ}{\delta }}_{2}$	0.04893^{4}^	0.05202^{5}^	0.04845^{2}^	0.04769^{1}^	0.05321^{6}^	0.04868^{3}^	0.05521^{7}^	0.06699^{8}^
	*MSE*	${\overset{ˆ}{\delta }}_{1}$	0.06252^{6}^	0.07937^{7}^	0.04164^{3}^	0.03382^{1}^	0.09505^{8}^	0.04877^{5}^	0.04579^{4}^	0.03693^{2}^
		${\overset{ˆ}{\delta }}_{2}$	0.00386^{3}^	0.00436^{5}^	0.00387^{4}^	0.00354^{1}^	0.00479^{6}^	0.00384^{2}^	0.00499^{7}^	0.00703^{8}^
	*MRE*	${\overset{ˆ}{\delta }}_{1}$	0.19640^{6}^	0.22756^{7}^	0.15952^{2}^	0.15616^{1}^	0.24124^{8}^	0.17903^{5}^	0.17122^{4}^	0.16120^{3}^
		${\overset{ˆ}{\delta }}_{2}$	0.09786^{4}^	0.10404^{5}^	0.09689^{2}^	0.09538^{1}^	0.10641^{6}^	0.09736^{3}^	0.11043^{7}^	0.13398^{8}^
	∑*Ranks*		29^{4}^	36^{7}^	15^{2}^	6^{1}^	42^{8}^	23^{3}^	33^{6}^	32^{5}^

80	|*BIAS*|	${\overset{ˆ}{\delta }}_{1}$	0.14421^{6}^	0.16973^{7}^	0.11641^{1}^	0.11707^{2}^	0.16994^{8}^	0.13475^{5}^	0.12334^{4}^	0.11846^{3}^
		${\overset{ˆ}{\delta }}_{2}$	0.03880^{4}^	0.04142^{5}^	0.03826^{2}^	0.03716^{1}^	0.04200^{6}^	0.03853^{3}^	0.04467^{7}^	0.05280^{8}^
	*MSE*	${\overset{ˆ}{\delta }}_{1}$	0.03649^{6}^	0.04750^{7}^	0.02362^{3}^	0.02070^{1}^	0.05062^{8}^	0.02959^{5}^	0.02516^{4}^	0.02160^{2}^
		${\overset{ˆ}{\delta }}_{2}$	0.00240^{4}^	0.00271^{5}^	0.00238^{3}^	0.00214^{1}^	0.00283^{6}^	0.00236^{2}^	0.00323^{7}^	0.00436^{8}^
	*MRE*	${\overset{ˆ}{\delta }}_{1}$	0.15180^{6}^	0.17866^{7}^	0.12254^{1}^	0.12323^{2}^	0.17888^{8}^	0.14184^{5}^	0.12983^{4}^	0.12469^{3}^
		${\overset{ˆ}{\delta }}_{2}$	0.07759^{4}^	0.08283^{5}^	0.07653^{2}^	0.07433^{1}^	0.08400^{6}^	0.07707^{3}^	0.08934^{7}^	0.10560^{8}^
	∑*Ranks*		30^{4}^	36^{7}^	12^{2}^	8^{1}^	42^{8}^	23^{3}^	33^{6}^	32^{5}^

120	|*BIAS*|	${\overset{ˆ}{\delta }}_{1}$	0.11428^{6}^	0.13664^{7}^	0.09311^{1}^	0.09414^{2}^	0.13762^{8}^	0.10913^{5}^	0.10206^{4}^	0.09587^{3}^
		${\overset{ˆ}{\delta }}_{2}$	0.03226^{4}^	0.03335^{5}^	0.03090^{2}^	0.03085^{1}^	0.03372^{6}^	0.03141^{3}^	0.03549^{7}^	0.04333^{8}^
	*MSE*	${\overset{ˆ}{\delta }}_{1}$	0.02188^{6}^	0.03019^{7}^	0.01459^{3}^	0.01359^{1}^	0.03178^{8}^	0.01936^{5}^	0.01717^{4}^	0.01441^{2}^
		${\overset{ˆ}{\delta }}_{2}$	0.00166^{4}^	0.00178^{5}^	0.00153^{2}^	0.00147^{1}^	0.00182^{6}^	0.00157^{3}^	0.00203^{7}^	0.00297^{8}^
	*MRE*	${\overset{ˆ}{\delta }}_{1}$	0.12029^{6}^	0.14384^{7}^	0.09801^{1}^	0.09909^{2}^	0.14486^{8}^	0.11487^{5}^	0.10743^{4}^	0.10092^{3}^
		${\overset{ˆ}{\delta }}_{2}$	0.06452^{4}^	0.06670^{5}^	0.06179^{2}^	0.06170^{1}^	0.06744^{6}^	0.06282^{3}^	0.07098^{7}^	0.08666^{8}^
	∑*Ranks*		30^{4}^	36^{7}^	11^{2}^	8^{1}^	42^{8}^	24^{3}^	33^{6}^	32^{5}^

200	|*BIAS*|	${\overset{ˆ}{\delta }}_{1}$	0.08764^{6}^	0.10235^{7}^	0.06800^{1}^	0.07225^{3}^	0.10486^{8}^	0.08285^{5}^	0.07874^{4}^	0.07106^{2}^
		${\overset{ˆ}{\delta }}_{2}$	0.02418^{4}^	0.02567^{5}^	0.02349^{2}^	0.02345^{1}^	0.02589^{6}^	0.02405^{3}^	0.02762^{7}^	0.03324^{8}^
	*MSE*	${\overset{ˆ}{\delta }}_{1}$	0.01247^{6}^	0.01703^{7}^	0.00768^{1}^	0.00801^{3}^	0.01826^{8}^	0.01106^{5}^	0.01005^{4}^	0.00788^{2}^
		${\overset{ˆ}{\delta }}_{2}$	0.00095^{4}^	0.00104^{5}^	0.00087^{2}^	0.00086^{1}^	0.00107^{6}^	0.00092^{3}^	0.00122^{7}^	0.00174^{8}^
	*MRE*	${\overset{ˆ}{\delta }}_{1}$	0.09225^{6}^	0.10774^{7}^	0.07158^{1}^	0.07606^{3}^	0.11038^{8}^	0.08721^{5}^	0.08289^{4}^	0.07480^{2}^
		${\overset{ˆ}{\delta }}_{2}$	0.04837^{4}^	0.05134^{5}^	0.04698^{2}^	0.04690^{1}^	0.05179^{6}^	0.04810^{3}^	0.05525^{7}^	0.06649^{8}^
	∑*Ranks*		30^{4.5}^	36^{7}^	9^{1}^	12^{2}^	42^{8}^	24^{3}^	33^{6}^	30^{4.5}^

300	|*BIAS*|	${\overset{ˆ}{\delta }}_{1}$	0.06988^{6}^	0.08546^{7}^	0.05636^{1}^	0.05751^{2}^	0.08691^{8}^	0.06780^{5}^	0.06432^{4}^	0.05820^{3}^
		${\overset{ˆ}{\delta }}_{2}$	0.01968^{4}^	0.02091^{5}^	0.01914^{2}^	0.01905^{1}^	0.02116^{6}^	0.01944^{3}^	0.02201^{7}^	0.02792^{8}^
	*MSE*	${\overset{ˆ}{\delta }}_{1}$	0.00783^{6}^	0.01149^{7}^	0.00514^{2}^	0.00507^{1}^	0.01230^{8}^	0.00731^{5}^	0.00670^{4}^	0.00524^{3}^
		${\overset{ˆ}{\delta }}_{2}$	0.00062^{4}^	0.00069^{5}^	0.00058^{2}^	0.00056^{1}^	0.00072^{6}^	0.00060^{3}^	0.00076^{7}^	0.00123^{8}^
	*MRE*	${\overset{ˆ}{\delta }}_{1}$	0.07356^{6}^	0.08996^{7}^	0.05932^{1}^	0.06054^{2}^	0.09149^{8}^	0.07137^{5}^	0.06771^{4}^	0.06126^{3}^
		${\overset{ˆ}{\delta }}_{2}$	0.03936^{4}^	0.04183^{5}^	0.03829^{2}^	0.03809^{1}^	0.04232^{6}^	0.03888^{3}^	0.04403^{7}^	0.05584^{8}^
	∑*Ranks*		30^{4}^	36^{7}^	10^{2}^	8^{1}^	42^{8}^	24^{3}^	33^{5.5}^	33^{5.5}^

**Table 4:** Simulation results for $\Psi ={\left(\delta_{1}=1.56,\delta_{2}=0.87\right)}^{⊺}$.

*n*	Est.	Est. Par.	WLS	OLS	ML	MPS	CVM	AD	RAD	PC
20	|*BIAS*|	${\overset{ˆ}{\delta }}_{1}$	0.37719^{6}^	0.40647^{7}^	0.34881^{5}^	0.32386^{2}^	0.46402^{8}^	0.33802^{3}^	0.34694^{4}^	0.32364^{1}^
		${\overset{ˆ}{\delta }}_{2}$	0.15076^{5}^	0.15293^{6}^	0.14999^{4}^	0.13776^{1}^	0.16923^{8}^	0.14166^{2}^	0.16078^{7}^	0.14700^{3}^
	*MSE*	${\overset{ˆ}{\delta }}_{1}$	0.26581^{6}^	0.29453^{7}^	0.22688^{5}^	0.16163^{1}^	0.42770^{8}^	0.19746^{3}^	0.22286^{4}^	0.16626^{2}^
		${\overset{ˆ}{\delta }}_{2}$	0.03931^{5}^	0.03967^{6}^	0.03920^{4}^	0.02920^{1}^	0.05267^{8}^	0.03399^{2}^	0.04647^{7}^	0.03434^{3}^
	*MRE*	${\overset{ˆ}{\delta }}_{1}$	0.24179^{6}^	0.26055^{7}^	0.22359^{5}^	0.20760^{2}^	0.29745^{8}^	0.21668^{3}^	0.22240^{4}^	0.20746^{1}^
		${\overset{ˆ}{\delta }}_{2}$	0.15869^{5}^	0.16098^{6}^	0.15789^{4}^	0.14501^{1}^	0.17814^{8}^	0.14911^{2}^	0.16924^{7}^	0.15473^{3}^
	∑*Ranks*		33^{5.5}^	39^{7}^	27^{4}^	8^{1}^	48^{8}^	15^{3}^	33^{5.5}^	13^{2}^

50	|*BIAS*|	${\overset{ˆ}{\delta }}_{1}$	0.21906^{6}^	0.25023^{7}^	0.20022^{3}^	0.19854^{2}^	0.26193^{8}^	0.21028^{5}^	0.20627^{4}^	0.19474^{1}^
		${\overset{ˆ}{\delta }}_{2}$	0.09024^{4}^	0.09948^{7}^	0.08932^{3}^	0.08718^{1}^	0.09836^{6}^	0.08726^{2}^	0.09988^{8}^	0.09232^{5}^
	*MSE*	${\overset{ˆ}{\delta }}_{1}$	0.08092^{6}^	0.10372^{7}^	0.06944^{3}^	0.05857^{1}^	0.11784^{8}^	0.07194^{4}^	0.07201^{5}^	0.05970^{2}^
		${\overset{ˆ}{\delta }}_{2}$	0.01307^{3}^	0.01623^{7}^	0.01327^{4}^	0.01152^{1}^	0.01598^{6}^	0.01214^{2}^	0.01677^{8}^	0.01329^{5}^
	*MRE*	${\overset{ˆ}{\delta }}_{1}$	0.14042^{6}^	0.16040^{7}^	0.12834^{3}^	0.12727^{2}^	0.16790^{8}^	0.13479^{5}^	0.13223^{4}^	0.12483^{1}^
		${\overset{ˆ}{\delta }}_{2}$	0.09499^{4}^	0.10471^{7}^	0.09402^{3}^	0.09177^{1}^	0.10354^{6}^	0.09186^{2}^	0.10514^{8}^	0.09718^{5}^
	∑*Ranks*		29^{5}^	42^{7.5}^	19^{2.5}^	8^{1}^	42^{7.5}^	20^{4}^	37^{6}^	19^{2.5}^

80	|*BIAS*|	${\overset{ˆ}{\delta }}_{1}$	0.17123^{6}^	0.19550^{8}^	0.14959^{1}^	0.15233^{2}^	0.19376^{7}^	0.16336^{5}^	0.16309^{4}^	0.15494^{3}^
		${\overset{ˆ}{\delta }}_{2}$	0.07042^{4}^	0.07535^{6}^	0.06754^{2}^	0.06717^{1}^	0.07719^{8}^	0.06965^{3}^	0.07677^{7}^	0.07439^{5}^
	*MSE*	${\overset{ˆ}{\delta }}_{1}$	0.04803^{6}^	0.06235^{7}^	0.03768^{3}^	0.03479^{1}^	0.06263^{8}^	0.04273^{4}^	0.04330^{5}^	0.03653^{2}^
		${\overset{ˆ}{\delta }}_{2}$	0.00802^{4}^	0.00907^{6}^	0.00741^{2}^	0.00702^{1}^	0.00954^{8}^	0.00772^{3}^	0.00934^{7}^	0.00877^{5}^
	*MRE*	${\overset{ˆ}{\delta }}_{1}$	0.10976^{6}^	0.12532^{8}^	0.09589^{1}^	0.09765^{2}^	0.12421^{7}^	0.10472^{5}^	0.10454^{4}^	0.09932^{3}^
		${\overset{ˆ}{\delta }}_{2}$	0.07413^{4}^	0.07932^{6}^	0.07109^{2}^	0.07071^{1}^	0.08125^{8}^	0.07332^{3}^	0.08081^{7}^	0.07831^{5}^
	∑*Ranks*		30^{5}^	41^{7}^	11^{2}^	8^{1}^	46^{8}^	23^{3.5}^	34^{6}^	23^{3.5}^

120	|*BIAS*|	${\overset{ˆ}{\delta }}_{1}$	0.13576^{6}^	0.15927^{8}^	0.12021^{1}^	0.12529^{3}^	0.15760^{7}^	0.13326^{5}^	0.13002^{4}^	0.12303^{2}^
		${\overset{ˆ}{\delta }}_{2}$	0.05661^{3}^	0.06305^{8}^	0.05626^{2}^	0.05540^{1}^	0.06268^{7}^	0.05764^{4}^	0.06212^{6}^	0.06084^{5}^
	*MSE*	${\overset{ˆ}{\delta }}_{1}$	0.02976^{6}^	0.04076^{7}^	0.02357^{2}^	0.02380^{3}^	0.04118^{8}^	0.02827^{5}^	0.02715^{4}^	0.02342^{1}^
		${\overset{ˆ}{\delta }}_{2}$	0.00514^{3}^	0.00624^{7}^	0.00505^{2}^	0.00472^{1}^	0.00629^{8}^	0.00522^{4}^	0.00615^{6}^	0.00575^{5}^
	*MRE*	${\overset{ˆ}{\delta }}_{1}$	0.08702^{6}^	0.10210^{8}^	0.07706^{1}^	0.08031^{3}^	0.10103^{7}^	0.08542^{5}^	0.08334^{4}^	0.07887^{2}^
		${\overset{ˆ}{\delta }}_{2}$	0.05959^{3}^	0.06637^{8}^	0.05922^{2}^	0.05831^{1}^	0.06598^{7}^	0.06067^{4}^	0.06539^{6}^	0.06405^{5}^
	∑*Ranks*		27^{4.5}^	46^{8}^	10^{1}^	12^{2}^	44^{7}^	27^{4.5}^	30^{6}^	20^{3}^

200	|*BIAS*|	${\overset{ˆ}{\delta }}_{1}$	0.10389^{6}^	0.12124^{7}^	0.09213^{1}^	0.09359^{2}^	0.12404^{8}^	0.10254^{5}^	0.10071^{4}^	0.09462^{3}^
		${\overset{ˆ}{\delta }}_{2}$	0.04365^{4}^	0.04615^{5}^	0.04313^{2}^	0.04260^{1}^	0.04861^{8}^	0.04343^{3}^	0.04762^{7}^	0.04661^{6}^
	*MSE*	${\overset{ˆ}{\delta }}_{1}$	0.01730^{6}^	0.02351^{7}^	0.01386^{3}^	0.01335^{1}^	0.02493^{8}^	0.01673^{5}^	0.01615^{4}^	0.01376^{2}^
		${\overset{ˆ}{\delta }}_{2}$	0.00302^{4}^	0.00339^{5}^	0.00296^{2}^	0.00282^{1}^	0.00377^{8}^	0.00300^{3}^	0.00357^{7}^	0.00342^{6}^
	*MRE*	${\overset{ˆ}{\delta }}_{1}$	0.06660^{6}^	0.07772^{7}^	0.05906^{1}^	0.05999^{2}^	0.07952^{8}^	0.06573^{5}^	0.06456^{4}^	0.06066^{3}^
		${\overset{ˆ}{\delta }}_{2}$	0.04594^{4}^	0.04858^{5}^	0.04540^{2}^	0.04484^{1}^	0.05117^{8}^	0.04571^{3}^	0.05013^{7}^	0.04907^{6}^
	∑*Ranks*		30^{5}^	36^{7}^	11^{2}^	8^{1}^	48^{8}^	24^{3}^	33^{6}^	26^{4}^

300	|*BIAS*|	${\overset{ˆ}{\delta }}_{1}$	0.08349^{5}^	0.09822^{7}^	0.07395^{1}^	0.07552^{2}^	0.10021^{8}^	0.08331^{4}^	0.08432^{6}^	0.07692^{3}^
		${\overset{ˆ}{\delta }}_{2}$	0.03653^{4}^	0.03867^{6}^	0.03465^{2}^	0.03443^{1}^	0.03881^{7}^	0.03638^{3}^	0.03912^{8}^	0.03858^{5}^
	*MSE*	${\overset{ˆ}{\delta }}_{1}$	0.01112^{5}^	0.01539^{7}^	0.00876^{1}^	0.00884^{2}^	0.01606^{8}^	0.01096^{4}^	0.01125^{6}^	0.00908^{3}^
		${\overset{ˆ}{\delta }}_{2}$	0.00209^{4}^	0.00237^{6}^	0.00191^{2}^	0.00184^{1}^	0.00241^{7}^	0.00206^{3}^	0.00243^{8}^	0.00233^{5}^
	*MRE*	${\overset{ˆ}{\delta }}_{1}$	0.05352^{5}^	0.06296^{7}^	0.04740^{1}^	0.04841^{2}^	0.06424^{8}^	0.05341^{4}^	0.05405^{6}^	0.04931^{3}^
		${\overset{ˆ}{\delta }}_{2}$	0.03845^{4}^	0.04071^{6}^	0.03647^{2}^	0.03624^{1}^	0.04086^{7}^	0.03829^{3}^	0.04118^{8}^	0.04061^{5}^
	∑*Ranks*		27^{5}^	39^{6}^	9^{1.5}^	9^{1.5}^	45^{8}^	21^{3}^	42^{7}^	24^{4}^

**Table 5:** Simulation results for $\Psi ={\left(\delta_{1}=2.21,\delta_{2}=1.39\right)}^{⊺}$.

*n*	Est.	Est. Par.	WLS	OLS	ML	MPS	CVM	AD	RAD	PC
20	|*BIAS*|	${\overset{ˆ}{\delta }}_{1}$	0.45439^{6}^	0.48754^{7}^	0.43302^{4}^	0.40323^{2}^	0.55262^{8}^	0.41430^{3}^	0.44715^{5}^	0.39329^{1}^
		${\overset{ˆ}{\delta }}_{2}$	0.24317^{4}^	0.25791^{6}^	0.24838^{5}^	0.22892^{2}^	0.28498^{8}^	0.23410^{3}^	0.26550^{7}^	0.22091^{1}^
	*MSE*	${\overset{ˆ}{\delta }}_{1}$	0.35830^{5}^	0.42142^{7}^	0.35572^{4}^	0.24226^{2}^	0.60652^{8}^	0.30031^{3}^	0.38007^{6}^	0.23741^{1}^
		${\overset{ˆ}{\delta }}_{2}$	0.10077^{4}^	0.11360^{6}^	0.11131^{5}^	0.08098^{2}^	0.15046^{8}^	0.09253^{3}^	0.12723^{7}^	0.07673^{1}^
	*MRE*	${\overset{ˆ}{\delta }}_{1}$	0.20561^{6}^	0.22061^{7}^	0.19594^{4}^	0.18246^{2}^	0.25005^{8}^	0.18747^{3}^	0.20233^{5}^	0.17796^{1}^
		${\overset{ˆ}{\delta }}_{2}$	0.15588^{4}^	0.16533^{6}^	0.15922^{5}^	0.14674^{2}^	0.18268^{8}^	0.15006^{3}^	0.17019^{7}^	0.14161^{1}^
	∑*Ranks*		29^{5}^	39^{7}^	27^{4}^	12^{2}^	48^{8}^	18^{3}^	37^{6}^	6^{1}^

50	|*BIAS*|	${\overset{ˆ}{\delta }}_{1}$	0.26787^{6}^	0.29738^{7}^	0.23496^{1}^	0.24443^{3}^	0.30804^{8}^	0.25156^{4}^	0.26414^{5}^	0.24398^{2}^
		${\overset{ˆ}{\delta }}_{2}$	0.14734^{5}^	0.15552^{6}^	0.13993^{1}^	0.14025^{3}^	0.16362^{8}^	0.14203^{4}^	0.15823^{7}^	0.14009^{2}^
	*MSE*	${\overset{ˆ}{\delta }}_{1}$	0.11715^{5}^	0.14422^{7}^	0.09447^{3}^	0.09042^{1}^	0.16088^{8}^	0.10146^{4}^	0.11827^{6}^	0.09154^{2}^
		${\overset{ˆ}{\delta }}_{2}$	0.03519^{5}^	0.03898^{6}^	0.03220^{3}^	0.03026^{1}^	0.04462^{8}^	0.03259^{4}^	0.04178^{7}^	0.03070^{2}^
	*MRE*	${\overset{ˆ}{\delta }}_{1}$	0.12121^{6}^	0.13456^{7}^	0.10632^{1}^	0.11060^{3}^	0.13938^{8}^	0.11383^{4}^	0.11952^{5}^	0.11040^{2}^
		${\overset{ˆ}{\delta }}_{2}$	0.09445^{5}^	0.09970^{6}^	0.08970^{1}^	0.08991^{3}^	0.10489^{8}^	0.09104^{4}^	0.10143^{7}^	0.08980^{2}^
	∑*Ranks*		32^{5}^	39^{7}^	10^{1}^	14^{3}^	48^{8}^	24^{4}^	37^{6}^	12^{2}^

80	|*BIAS*|	${\overset{ˆ}{\delta }}_{1}$	0.20926^{6}^	0.23233^{7}^	0.18569^{2}^	0.19200^{3}^	0.23643^{8}^	0.19562^{4}^	0.20345^{5}^	0.18493^{1}^
		${\overset{ˆ}{\delta }}_{2}$	0.11578^{5}^	0.12239^{7}^	0.11086^{4}^	0.10987^{2}^	0.12534^{8}^	0.11074^{3}^	0.12204^{6}^	0.10720^{1}^
	*MSE*	${\overset{ˆ}{\delta }}_{1}$	0.07255^{6}^	0.08646^{7}^	0.05643^{3}^	0.05613^{2}^	0.09204^{8}^	0.06158^{4}^	0.06728^{5}^	0.05274^{1}^
		${\overset{ˆ}{\delta }}_{2}$	0.02202^{5}^	0.02396^{6}^	0.01983^{4}^	0.01843^{2}^	0.02564^{8}^	0.01972^{3}^	0.02415^{7}^	0.01797^{1}^
	*MRE*	${\overset{ˆ}{\delta }}_{1}$	0.09469^{6}^	0.10513^{7}^	0.08402^{2}^	0.08688^{3}^	0.10698^{8}^	0.08852^{4}^	0.09206^{5}^	0.08368^{1}^
		${\overset{ˆ}{\delta }}_{2}$	0.07422^{5}^	0.07845^{7}^	0.07107^{4}^	0.07043^{2}^	0.08035^{8}^	0.07099^{3}^	0.07823^{6}^	0.06872^{1}^
	∑*Ranks*		33^{5}^	41^{7}^	19^{3}^	14^{2}^	48^{8}^	21^{4}^	34^{6}^	6^{1}^

120	|*BIAS*|	${\overset{ˆ}{\delta }}_{1}$	0.16630^{6}^	0.18585^{7}^	0.14935^{1}^	0.15438^{3}^	0.18866^{8}^	0.16574^{5}^	0.16330^{4}^	0.15147^{2}^
		${\overset{ˆ}{\delta }}_{2}$	0.09403^{5}^	0.09833^{6}^	0.09196^{3}^	0.08917^{2}^	0.10174^{8}^	0.09258^{4}^	0.09969^{7}^	0.08803^{1}^
	*MSE*	${\overset{ˆ}{\delta }}_{1}$	0.04420^{6}^	0.05485^{7}^	0.03652^{2}^	0.03681^{3}^	0.05775^{8}^	0.04318^{5}^	0.04279^{4}^	0.03557^{1}^
		${\overset{ˆ}{\delta }}_{2}$	0.01422^{5}^	0.01537^{6}^	0.01342^{3}^	0.01230^{2}^	0.01673^{8}^	0.01351^{4}^	0.01577^{7}^	0.01217^{1}^
	*MRE*	${\overset{ˆ}{\delta }}_{1}$	0.07525^{6}^	0.08409^{7}^	0.06758^{1}^	0.06986^{3}^	0.08536^{8}^	0.07500^{5}^	0.07389^{4}^	0.06854^{2}^
		${\overset{ˆ}{\delta }}_{2}$	0.06027^{5}^	0.06303^{6}^	0.05895^{3}^	0.05716^{2}^	0.06522^{8}^	0.05935^{4}^	0.06390^{7}^	0.05643^{1}^
	∑*Ranks*		33^{5.5}^	39^{7}^	13^{2}^	15^{3}^	48^{8}^	27^{4}^	33^{5.5}^	8^{1}^

200	|*BIAS*|	${\overset{ˆ}{\delta }}_{1}$	0.12758^{5}^	0.14676^{7}^	0.11213^{1}^	0.11780^{3}^	0.14743^{8}^	0.12568^{4}^	0.12857^{6}^	0.11469^{2}^
		${\overset{ˆ}{\delta }}_{2}$	0.07122^{5}^	0.07684^{6}^	0.06740^{1}^	0.06920^{3}^	0.07761^{7}^	0.07015^{4}^	0.07802^{8}^	0.06841^{2}^
	*MSE*	${\overset{ˆ}{\delta }}_{1}$	0.02631^{5}^	0.03436^{7}^	0.02035^{1}^	0.02145^{3}^	0.03456^{8}^	0.02501^{4}^	0.02670^{6}^	0.02046^{2}^
		${\overset{ˆ}{\delta }}_{2}$	0.00807^{5}^	0.00942^{6}^	0.00712^{1}^	0.00737^{3}^	0.00958^{7}^	0.00775^{4}^	0.00968^{8}^	0.00732^{2}^
	*MRE*	${\overset{ˆ}{\delta }}_{1}$	0.05773^{5}^	0.06641^{7}^	0.05074^{1}^	0.05330^{3}^	0.06671^{8}^	0.05687^{4}^	0.05818^{6}^	0.05190^{2}^
		${\overset{ˆ}{\delta }}_{2}$	0.04565^{5}^	0.04926^{6}^	0.04320^{1}^	0.04436^{3}^	0.04975^{7}^	0.04497^{4}^	0.05001^{8}^	0.04385^{2}^
	∑*Ranks*		30^{5}^	39^{6}^	6^{1}^	18^{3}^	45^{8}^	24^{4}^	42^{7}^	12^{2}^

300	|*BIAS*|	${\overset{ˆ}{\delta }}_{1}$	0.10432^{5}^	0.11965^{7}^	0.09240^{1}^	0.09638^{3}^	0.12119^{8}^	0.10052^{4}^	0.10478^{6}^	0.09352^{2}^
		${\overset{ˆ}{\delta }}_{2}$	0.05813^{5}^	0.06280^{7}^	0.05554^{1}^	0.05612^{2}^	0.06414^{8}^	0.05716^{4}^	0.06268^{6}^	0.05624^{3}^
	*MSE*	${\overset{ˆ}{\delta }}_{1}$	0.01729^{5.5}^	0.02227^{7}^	0.01346^{1}^	0.01443^{3}^	0.02341^{8}^	0.01585^{4}^	0.01729^{5.5}^	0.01364^{2}^
		${\overset{ˆ}{\delta }}_{2}$	0.00531^{5}^	0.00634^{7}^	0.00486^{1}^	0.00494^{2}^	0.00650^{8}^	0.00522^{4}^	0.00616^{6}^	0.00495^{3}^
	*MRE*	${\overset{ˆ}{\delta }}_{1}$	0.04720^{5}^	0.05414^{7}^	0.04181^{1}^	0.04361^{3}^	0.05484^{8}^	0.04548^{4}^	0.04741^{6}^	0.04232^{2}^
		${\overset{ˆ}{\delta }}_{2}$	0.03726^{5}^	0.04026^{7}^	0.03560^{1}^	0.03598^{2}^	0.04112^{8}^	0.03664^{4}^	0.04018^{6}^	0.03605^{3}^
	∑*Ranks*		30.5^{5}^	42^{7}^	6^{1}^	15^{2.5}^	48^{8}^	24^{4}^	35.5^{6}^	15^{2.5}^

**Table 6:** Simulation results for $\Psi ={\left(\delta_{1}=3.00,\delta_{2}=4.00\right)}^{⊺}$.

*n*	Est.	Est. Par.	WLS	OLS	ML	MPS	CVM	AD	RAD	PC
20	|*BIAS*|	${\overset{ˆ}{\delta }}_{1}$	0.56148^{5}^	0.59676^{7}^	0.52677^{4}^	0.50398^{2}^	0.66778^{8}^	0.51367^{3}^	0.57456^{6}^	0.48710^{1}^
		${\overset{ˆ}{\delta }}_{2}$	0.47667^{5}^	0.50205^{6}^	0.46958^{4}^	0.43125^{2}^	0.55551^{8}^	0.45391^{3}^	0.51600^{7}^	0.42827^{1}^
	*MSE*	${\overset{ˆ}{\delta }}_{1}$	0.56182^{5}^	0.62115^{7}^	0.51586^{4}^	0.37834^{2}^	0.89048^{8}^	0.44820^{3}^	0.61001^{6}^	0.36857^{1}^
		${\overset{ˆ}{\delta }}_{2}$	0.39363^{4}^	0.43224^{6}^	0.39592^{5}^	0.28345^{1}^	0.60109^{8}^	0.34834^{3}^	0.48721^{7}^	0.28688^{2}^
	*MRE*	${\overset{ˆ}{\delta }}_{1}$	0.18716^{5}^	0.19892^{7}^	0.17559^{4}^	0.16799^{2}^	0.22259^{8}^	0.17122^{3}^	0.19152^{6}^	0.16237^{1}^
		${\overset{ˆ}{\delta }}_{2}$	0.15889^{5}^	0.16735^{6}^	0.15653^{4}^	0.14375^{2}^	0.18517^{8}^	0.15130^{3}^	0.17200^{7}^	0.14276^{1}^
	∑*Ranks*		29^{5}^	39^{6.5}^	25^{4}^	11^{2}^	48^{8}^	18^{3}^	39^{6.5}^	7^{1}^

50	|*BIAS*|	${\overset{ˆ}{\delta }}_{1}$	0.33217^{6}^	0.35979^{7}^	0.29961^{2}^	0.30913^{3}^	0.37751^{8}^	0.31557^{4}^	0.32381^{5}^	0.29699^{1}^
		${\overset{ˆ}{\delta }}_{2}$	0.28752^{5}^	0.30517^{7}^	0.27156^{3}^	0.27078^{2}^	0.31593^{8}^	0.27537^{4}^	0.29692^{6}^	0.26495^{1}^
	*MSE*	${\overset{ˆ}{\delta }}_{1}$	0.18095^{6}^	0.21223^{7}^	0.15277^{3}^	0.14196^{2}^	0.24637^{8}^	0.15857^{4}^	0.17508^{5}^	0.13711^{1}^
		${\overset{ˆ}{\delta }}_{2}$	0.13516^{5}^	0.15169^{7}^	0.12234^{4}^	0.11132^{2}^	0.16855^{8}^	0.12137^{3}^	0.14610^{6}^	0.10897^{1}^
	*MRE*	${\overset{ˆ}{\delta }}_{1}$	0.11072^{6}^	0.11993^{7}^	0.09987^{2}^	0.10304^{3}^	0.12584^{8}^	0.10519^{4}^	0.10794^{5}^	0.09900^{1}^
		${\overset{ˆ}{\delta }}_{2}$	0.09584^{5}^	0.10172^{7}^	0.09052^{3}^	0.09026^{2}^	0.10531^{8}^	0.09179^{4}^	0.09897^{6}^	0.08832^{1}^
	∑*Ranks*		33^{5.5}^	42^{7}^	17^{3}^	14^{2}^	48^{8}^	23^{4}^	33^{5.5}^	6^{1}^

80	|*BIAS*|	${\overset{ˆ}{\delta }}_{1}$	0.25630^{6}^	0.28765^{7}^	0.23347^{2}^	0.24410^{3}^	0.28969^{8}^	0.24799^{4}^	0.25569^{5}^	0.23251^{1}^
		${\overset{ˆ}{\delta }}_{2}$	0.22281^{5}^	0.24050^{7}^	0.20992^{2}^	0.21455^{3}^	0.24590^{8}^	0.21912^{4}^	0.23302^{6}^	0.20767^{1}^
	*MSE*	${\overset{ˆ}{\delta }}_{1}$	0.10632^{6}^	0.13369^{7}^	0.08833^{2}^	0.08984^{3}^	0.13417^{8}^	0.09883^{4}^	0.10550^{5}^	0.08234^{1}^
		${\overset{ˆ}{\delta }}_{2}$	0.08034^{5}^	0.09311^{7}^	0.07147^{3}^	0.07048^{2}^	0.09622^{8}^	0.07722^{4}^	0.08867^{6}^	0.06654^{1}^
	*MRE*	${\overset{ˆ}{\delta }}_{1}$	0.08543^{6}^	0.09588^{7}^	0.07782^{2}^	0.08137^{3}^	0.09656^{8}^	0.08266^{4}^	0.08523^{5}^	0.07750^{1}^
		${\overset{ˆ}{\delta }}_{2}$	0.07427^{5}^	0.08017^{7}^	0.06997^{2}^	0.07152^{3}^	0.08197^{8}^	0.07304^{4}^	0.07767^{6}^	0.06922^{1}^
	∑*Ranks*		33^{5.5}^	42^{7}^	13^{2}^	17^{3}^	48^{8}^	24^{4}^	33^{5.5}^	6^{1}^

120	|*BIAS*|	${\overset{ˆ}{\delta }}_{1}$	0.20679^{5}^	0.23446^{7}^	0.18692^{1}^	0.19122^{3}^	0.23561^{8}^	0.19894^{4}^	0.20714^{6}^	0.19007^{2}^
		${\overset{ˆ}{\delta }}_{2}$	0.17849^{5}^	0.20057^{8}^	0.16963^{1}^	0.17147^{3}^	0.19876^{7}^	0.17447^{4}^	0.18953^{6}^	0.17083^{2}^
	*MSE*	${\overset{ˆ}{\delta }}_{1}$	0.06885^{5}^	0.08877^{7}^	0.05607^{2}^	0.05605^{1}^	0.09139^{8}^	0.06291^{4}^	0.06944^{6}^	0.05624^{3}^
		${\overset{ˆ}{\delta }}_{2}$	0.05112^{5}^	0.06407^{7}^	0.04639^{3}^	0.04524^{1}^	0.06411^{8}^	0.04777^{4}^	0.05807^{6}^	0.04538^{2}^
	*MRE*	${\overset{ˆ}{\delta }}_{1}$	0.06893^{5}^	0.07815^{7}^	0.06231^{1}^	0.06374^{3}^	0.07854^{8}^	0.06631^{4}^	0.06905^{6}^	0.06336^{2}^
		${\overset{ˆ}{\delta }}_{2}$	0.05950^{5}^	0.06686^{8}^	0.05654^{1}^	0.05716^{3}^	0.06625^{7}^	0.05816^{4}^	0.06318^{6}^	0.05694^{2}^
	∑*Ranks*		30^{5}^	44^{7}^	9^{1}^	14^{3}^	46^{8}^	24^{4}^	36^{6}^	13^{2}^

200	|*BIAS*|	${\overset{ˆ}{\delta }}_{1}$	0.15852^{5}^	0.17759^{7}^	0.13972^{1}^	0.14864^{3}^	0.18015^{8}^	0.15504^{4}^	0.16314^{6}^	0.14622^{2}^
		${\overset{ˆ}{\delta }}_{2}$	0.13846^{5}^	0.15098^{7}^	0.12757^{1}^	0.13227^{3}^	0.15100^{8}^	0.13585^{4}^	0.15010^{6}^	0.13118^{2}^
	*MSE*	${\overset{ˆ}{\delta }}_{1}$	0.04003^{5}^	0.05044^{7}^	0.03163^{1}^	0.03405^{3}^	0.05164^{8}^	0.03843^{4}^	0.04284^{6}^	0.03323^{2}^
		${\overset{ˆ}{\delta }}_{2}$	0.02999^{5}^	0.03628^{7}^	0.02590^{1}^	0.02706^{3}^	0.03664^{8}^	0.02929^{4}^	0.03563^{6}^	0.02664^{2}^
	*MRE*	${\overset{ˆ}{\delta }}_{1}$	0.05284^{5}^	0.05920^{7}^	0.04657^{1}^	0.04955^{3}^	0.06005^{8}^	0.05168^{4}^	0.05438^{6}^	0.04874^{2}^
		${\overset{ˆ}{\delta }}_{2}$	0.04615^{5}^	0.05033^{7.5}^	0.04252^{1}^	0.04409^{3}^	0.05033^{7.5}^	0.04528^{4}^	0.05003^{6}^	0.04373^{2}^
	∑*Ranks*		30^{5}^	42.5^{7}^	6^{1}^	18^{3}^	47.5^{8}^	24^{4}^	36^{6}^	12^{2}^

300	|*BIAS*|	${\overset{ˆ}{\delta }}_{1}$	0.12615^{4}^	0.14538^{7}^	0.11452^{1}^	0.12018^{3}^	0.14655^{8}^	0.12644^{5}^	0.12852^{6}^	0.11846^{2}^
		${\overset{ˆ}{\delta }}_{2}$	0.11179^{5}^	0.12338^{7}^	0.10516^{1}^	0.10693^{3}^	0.12457^{8}^	0.11168^{4}^	0.11616^{6}^	0.10594^{2}^
	*MSE*	${\overset{ˆ}{\delta }}_{1}$	0.02539^{4}^	0.03376^{7}^	0.02068^{1}^	0.02195^{2}^	0.03433^{8}^	0.02547^{5}^	0.02640^{6}^	0.02198^{3}^
		${\overset{ˆ}{\delta }}_{2}$	0.01990^{5}^	0.02417^{7}^	0.01744^{1}^	0.01760^{2}^	0.02459^{8}^	0.01959^{4}^	0.02167^{6}^	0.01761^{3}^
	*MRE*	${\overset{ˆ}{\delta }}_{1}$	0.04205^{4}^	0.04846^{7}^	0.03817^{1}^	0.04006^{3}^	0.04885^{8}^	0.04215^{5}^	0.04284^{6}^	0.03949^{2}^
		${\overset{ˆ}{\delta }}_{2}$	0.03726^{5}^	0.04113^{7}^	0.03505^{1}^	0.03564^{3}^	0.04152^{8}^	0.03723^{4}^	0.03872^{6}^	0.03531^{2}^
	∑*Ranks*		27^{4.5}^	42^{7}^	6^{1}^	16^{3}^	48^{8}^	27^{4.5}^	36^{6}^	14^{2}^

**Table 7:** The partial and overall ranks of the estimators.

**Ψ**^⊺^	*n*	**WLSE**	**OLSE**	**MLE**	**MPSE**	**CVME**	**ADE**	**RADE**	**PCE**
	20	5	7	2	1	8	3	6	4
	50	4	7	2	1	8	3	6	5
(*δ*_1_ = 0.10,*δ*_2_ = 0.05)	80	4	7	2	1	8	3	6	5
	120	4	7	2	1	8	3	6	5
	200	4	7	2	1	8	3	6	5
	300	4	7	2	1	8	3	5.5	5.5

	20	6	7	2.5	1	8	2.5	4.5	4.5
	50	5	7	2	1	8	3	6	4
(*δ*_1_ = 0.10,*δ*_2_ = 0.45)	80	4	7.5	2	1	7.5	3	6	5
	120	3.5	7	2	1	8	3.5	6	5
	200	5	7	1	2	8	3	6	4
	300	3.5	7.5	2	1	7.5	3.5	6	5

	20	5	7	2	1	8	3	5	5
	50	5	7	2	1	8	3	6	4
(*δ*_1_ = 0.10,*δ*_2_ = 0.87)	80	5	7	2	1	8	3	6	4
	120	5	7.5	2	1	7.5	3	6	4
	200	5	7	2	1	8	3	6	4
	300	3.5	7	1	2	8	3.5	6	5

	20	5.5	7	2.5	1	8	2.5	5.5	4
	50	5	7	2	1	8	3	6	4
(*δ*1 = 0.10,*δ*_2_ = 1.39)	80	5	7	2	1	8	3	6	4
	120	3.5	7	1	2	8	3.5	6	5
	200	5	7	2	1	8	3	6	4
	300	4.5	7	1	2	8	3	6	4.5

	20	5	7	3	1	8	2	5	5
	50	4.5	7	2	1	8	3	6	4.5
(*δ*_1_ = 0.10,*δ*_2_ = 2.15)	80	4.5	7	2	1	8	3	6	4.5
	120	3.5	7	2	1	8	3.5	6	5
	200	5	7	1	2	8	3	6	4
	300	3	7	1	2	8	4	6	5

	20	6	7	3	1	8	2	4.5	4.5
	50	5	7	2	1	8	3	6	4
(*δ*_1_ = 0.10,*δ*_2_ = 4.00)	80	5	7	2	1	8	3	6	4
	120	5	7	2	1	8	3	6	4
	200	4	7	1	2	8	3	6	5
	300	3.5	7	1	2	8	3.5	6	5

	20	4	7	3	1	8	2	6	5
	50	3.5	7	2	1	8	3.5	6	5
(*δ*_1_ = 0.50,*δ*_2_ = 0.05)	80	5	7	2	1	8	3	6	4
	120	4	7	2	1	8	3	6	5
	200	4	7	1	2	8	3	6	5
	300	4	7	1	2	8	3	5.5	5.5

	20	5	7	2	1	8	3	6	4
	50	4	7	2	1	8	3	6	5
(*δ*_1_ = 0.50,*δ*_2_ = 0.45)	80	5	7	2	1	8	3	6	4
	120	5	7	1	2	8	3	6	4
	200	4	7	1	2	8	3	6	5
	300	5	7	1	2	8	3	6	4

	20	3.5	7	3.5	1	8	2	6	5
	50	4.5	7	2	1	8	3	6	4.5
(*δ*_1_ = 0.50,*δ*_2_ = 0.87)	80	5	7	1	2	8	3	6	4
	120	4	7	1	2	8	3	6	5
	200	3.5	7	1	2	8	3.5	6	5
	300	4	7.5	2	1	7.5	3	5.5	5.5

	20	5	7	4	1	8	2	6	3
	50	4	7	2	1	8	3	5.5	5.5
(*δ*_1_ = 0.50,*δ*_2_ = 1.39)	80	4	7	2	1	8	3	6	5
	120	4	7	2	1	8	3	6	5
	200	3	7.5	1	2	7.5	4	5.5	5.5
	300	3	7	1	2	8	4	5.5	5.5
	20	5	7	4	1	8	2	6	3
	50	5	7	2	1	8	4	6	3
(*δ*_1_ = 0.50,*δ*_2_ = 2.15)	80	4	7	2	1	8	3	5	6
	120	5	6.5	1.5	1.5	8	3	6.5	4
	200	4	8	2	1	7	3	5.5	5.5
	300	3.5	8	1.5	1.5	7	3.5	5.5	5.5

	20	5	7	4	1	8	3	6	2
	50	5	7	2	1	8	3	6	4
(*δ*_1_ = 0.50,*δ*_2_ = 4.00)	80	5	7	2	1	8	3	6	4
	120	5	7	1	2	8	4	6	3
	200	5	7	1	2	8	3	6	4
	300	5	7	1.5	1.5	8	3.5	6	3.5

	20	5	7	2	1	8	3	5	5
	50	5	7	2	1	8	3	6	4
(*δ*_1_ = 0.95,*δ*_2_ = 0.05)	80	5	7	2	1	8	3	6	4
	120	4	7	2	1	8	3	6	5
	200	5	7	1	2	8	4	6	3
	300	5	7	2	1	8	3	6	4

	20	3.5	7	3.5	1	8	2	5.5	5.5
	50	4	7	2	1	8	3	6	5
(*δ*_1_ = 0.95,*δ*_2_ = 0.45)	80	4	7	2	1	8	3	6	5
	120	4	7	2	1	8	3	6	5
	200	4.5	7	1	2	8	3	6	4.5
	300	4	7	2	1	8	3	5.5	5.5

	20	5	7	4	1	8	2	6	3
	50	4	7	2	1	8	3	6	5
(*δ*_1_ = 0.95,*δ*_2_ = 0.87)	80	5	7	1	2	8	3	6	4
	120	4	7	1	2	8	3	5.5	5.5
	200	4	7	1	2	8	3	5.5	5.5
	300	4	8	1	2	7	3	5.5	5.5

	20	5	7	4	1	8	3	6	2
	50	5	7	2	1	8	4	6	3
(*δ*_1_ = 0.95,*δ*_2_ = 1.39)	80	5	7	1	2	8	4	6	3
	120	5	7.5	1	2	7.5	4	6	3
	200	4.5	7	1	2	8	4.5	6	3
	300	4	8	1	2	7	3	6	5

	20	5	7	4	1	8	3	6	2
	50	5.5	7	2	1	8	3.5	5.5	3.5
(*δ*_1_ = 0.95,*δ*_2_ = 2.15)	80	5	7	2	1	8	4	6	3
	120	5	6.5	1.5	1.5	8	3.5	6.5	3.5
	200	4	8	1	2	6.5	5	6.5	3
	300	4	8	1	2	7	5	6	3

	20	5	7	4	2	8	3	6	1
	50	6	7	3	1.5	8	4	5	1.5
(*δ*_1_ = 0.95,*δ*_2_ = 4.00)	80	5.5	7	1	3	8	4	5.5	2
	120	5	7	1	2	8	4	6	3
	200	4	6.5	1	3	8	5	6.5	2
	300	5	7	1	3	8	4	6	2

	20	6	7	2	1	8	3	5	4
	50	5	7	2	1	8	3	6	4
(*δ*_1_ = 1.56,*δ*_2_ = 0.05)	80	5	7	2	1	8	3	6	4
	120	4.5	7	2	1	8	3	6	4.5
	200	4	8	1	2	7	3	6	5
	300	5	7	1	2	8	3	6	4

	20	5	7	3.5	1	8	2	6	3.5
	50	4	7	2	1	8	3	6	5
(*δ*_1_ = 1.56,*δ*_2_ = 0.45)	80	4	7	2	1	8	3	6	5
	120	4	7	1	2	8	3	6	5
	200	4	7	1	2	8	3	5.5	5.5
	300	4.5	7.5	1	2	7.5	3	6	4.5
	20	5.5	7	4	1	8	3	5.5	2
	50	5	7.5	2.5	1	7.5	4	6	2.5
(*δ*_1_ = 1.56,*δ*_2_ = 0.87)	80	5	7	2	1	8	3.5	6	3.5
	120	4.5	8	1	2	7	4.5	6	3
	200	5	7	2	1	8	3	6	4
	300	5	6	1.5	1.5	8	3	7	4

	20	5	7	4	2	8	3	6	1
	50	5	7	2	1	8	4	6	3
(*δ*_1_ = 1.56,*δ*_2_ = 1.39)	80	6	7	1	2	8	4	5	3
	120	5	7	1	3	8	4	6	2
	200	6	8	1	2.5	7	4	5	2.5
	300	5	7	1	2	8	3	6	4

	20	5	7	4	2	8	3	6	1
	50	5	7	2.5	2.5	8	4	6	1
(*δ*_1_ = 1.56,*δ*_2_ = 2.15)	80	5	7	2	3	8	4	6	1
	120	6	8	1	3	7	4	5	2
	200	5	6.5	3	2	8	4	6.5	1
	300	5	8	1	3	7	4	6	2

	20	5	7	4	2	8	3	6	1
	50	5	7	2.5	2.5	8	4	6	1
(*δ*_1_ = 1.56,*δ*_2_ = 4.00)	80	5	7	1	3	8	4	6	2
	120	5.5	8	3	2	7	4	5.5	1
	200	5.5	7	1	3	8	4	5.5	2
	300	5.5	7.5	1	3	7.5	4	5.5	2

	20	5.5	7	3	1	8	2	5.5	4
	50	4	7	2	1	8	3	6	5
(*δ*_1_ = 2.21,*δ*_2_ = 0.05)	80	4	7	2	1	8	3	6	5
	120	5	7	1	2	8	3	6	4
	200	4	7	1	2	8	3	6	5
	300	4	8	2	1	7	3	6	5

	20	5	7	4	1	8	2	6	3
	50	5	7	2	1	8	3	6	4
(*δ*_1_ = 2.21,*δ*_2_ = 0.45)	80	6	7	2	1	8	4	5	3
	120	5	7	1.5	1.5	8	3	6	4
	200	4.5	6	1	2	8	3	7	4.5
	300	5	7	2	1	8	3	4	6

	20	5	7	4	2	8	3	6	1
	50	5	7	4	1	8	3	6	2
(*δ*_1_ = 2.21,*δ*_2_ = 0.87)	80	5	7	1	2	8	4	6	3
	120	5	7	1	2	8	4	6	3
	200	5	7	1	2	8	3	6	4
	300	5	6	1	2	8	4	7	3

	20	5	7	4	2	8	3	6	1
	50	5	7	1	3	8	4	6	2
(*δ*_1_ = 2.21,*δ*_2_ = 1.39)	80	5	7	3	2	8	4	6	1
	120	5.5	7	2	3	8	4	5.5	1
	200	5	6	1	3	8	4	7	2
	300	5	7	1	2.5	8	4	6	2.5

	20	5	7	4	2	8	3	6	1
	50	5	7	3	2	8	4	6	1
(*δ*_1_ = 2.21,*δ*_2_ = 2.15)	80	5.5	7	2	3	8	4	5.5	1
	120	5	7	2	3	8	4	6	1
	200	5	7	1	3	8	4	6	2
	300	5	7	1.5	3	8	4	6	1.5

	20	5	7	4	2	8	3	6	1
	50	5	7	3	2	8	4	6	1
(*δ*_1_ = 2.21,*δ*_2_ = 4.00)	80	5.5	7	2	3	8	4	5.5	1
	120	5	7.5	1.5	3	7.5	4	6	1.5
	200	5	8	1	3	7	4	6	2
	300	5	7	1	3	8	4	6	2
	20	5.5	7	3	1	8	2	5.5	4
	50	5	7	2	1	8	3	6	4
(*δ*_1_ = 3.00,*δ*_2_ = 0.05)	80	5	7	2	1	8	3	6	4
	120	5	7	2	1	8	3	6	4
	200	4	7	2	1	8	3	6	5
	300	4.5	7	2	1	8	3	4.5	6

	20	4.5	7	4.5	1	8	3	6	2
	50	5	7	2	1	8	3.5	6	3.5
(*δ*_1_ = 3.00,*δ*_2_ = 0.45)	80	5	7	2	1	8	4	6	3
	120	5	7	1	2	8	3	6	4
	200	5	7	1	2	8	3	6	4
	300	5	6.5	1	2	8	3	6.5	4

	20	5	7	4	2	8	3	6	1
	50	5.5	7	2.5	2.5	8	4	5.5	1
(*δ*_1_ = 3.00,*δ*_2_ = 0.87)	80	5	7	1	2	8	4	6	3
	120	5	7	1	3	8	4	6	2
	200	5	7.5	1	2	7.5	4	6	3
	300	6	7	1	2	8	4	5	3

	20	5	7	4	2	8	3	6	1
	50	5	7	2	3	8	4	6	1
(*δ*_1_ = 3.00,*δ*_2_ = 1.39)	80	5	7	2	3	8	4	6	1
	120	5.5	7	1	3	8	4	5.5	2
	200	5	7	1	3	8	4	6	2
	300	5	7	1	3	8	4	6	2

	20	5	7	4	2	8	3	6	1
	50	5	7	2	3	8	4	6	1
(*δ*_1_ = 3.00,*δ*_2_ = 2.15)	80	5	7	1	3	8	4	6	2
	120	5	7	2	3	8	4	6	1
	200	5	7	1.5	3	8	4	6	1.5
	300	5	8	1.5	3	7	4	6	1.5

	20	5	6.5	4	2	8	3	6.5	1
	50	5.5	7	3	2	8	4	5.5	1
(*δ*_1_ = 3.00,*δ*_2_ = 4.00)	80	5.5	7	2	3	8	4	5.5	1
	120	5	7	1	3	8	4	6	2
	200	5	7	1	3	8	4	6	2
	300	4.5	7	1	3	8	4.5	6	2

∑*Ranks*		1025	1525.5	413	371	1707	720.5	1271	743
Overall Rank		5	7	2	1	8	3	6	4

Table 7 summarizes the partial and overall rank of the estimators. From Table 1, Table 2, Table 3, Table 4, Table 5, Table 6, Table 7, we observe that:

• The estimators $\delta_{1}$ and $\delta_{1}$ carry the property of consistency and tend to stable.
• For the NFWE distribution, the MPS and ML estimators have superior performances.

## 6 An application to the flood data

This section explores and demonstrates the applicability and superior performance of the NFWE distribution using an extreme value data. To establish the exceptional performance of the NFWE distribution over the other rival distributions, we examine a data set representing the maximum levels of the flood.

### 6.1 Description of the flood data set

Here, we provide the basic description of the flood data t that is considered to prove and establish the applicability of the NFWE distribution. The considered data set represents the maximum levels of the flood and is available at: https://data.world/datasets/flood. For interested readers, the data set is also provided in Appendix A.

Table 8 presents some useful key quantities of the flood data. From the key quantities, we can see the skewness is greater than 0, showing that the data is righted-skewed with a long right tail. Whereas, the value of kurtosis is 5.338 which ensures that the distribution of the flood data is Platykurtic. In Table 8, the terms $Q_{1}$, $Q_{2}$, and $Q_{3}$ represent the $1^{st}$ quartile, $2^{nd}$ quartile, and $3^{rd}$ quartile, respectively.

**Table 8:** The BMs of the flood data.

Min.	*Q*_1_	*Q*_2_	*Q*_3_	Mean	Max.	Skewness	Kurtosis	Variance	Range
0.20830	0.59140	0.97140	1.96980	1.47060	6.18760	1.61140	5.33860	1.59130	5.97930

**Figure 3:**
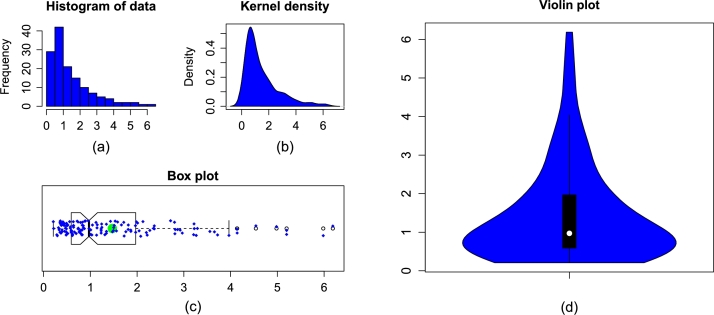
Certain key plots of the flood data set displaying (a) histogram, (b) kernel density, (c) box plot, and (d) violin plot.

We show the graphical illustration of the flood data set in Fig. 3(a-d). In this regard, the histogram and box plot of the flood data are sketched. The TTT plot of the flood data is also sketched. The plots, provided in Fig. 3(a-d), show the heavy-tailed characteristics of the flood data that experienced extreme observations.

### 6.2 Competing distributions

For the comparative purpose, the proposed NFWE distribution is applied in comparison with the two-parameter rival models and three-parameter rival models. The two-parameter distributions include the FWE and Weibull distributions. Whereas, the three-parameter competitive distributions include the Marshall-Olkin Weibull (MO-Weibull), exponentiated Weibull (E-Weibull), and Marshall-Olkin Weibull (MO-Weibull), and alpha power transformed Weibull (APT-Weibull) distributions. The PDFs of these two-parameter and three-parameter competitive models are given by

• Weibull distribution
  $$f(u)=\alpha \sigma u^{\alpha -1}e^{-\sigma u^{\alpha }},\;\;\;\;\;\;\;\;u>0,\alpha ,\sigma >0.$$
• E-Weibull distribution
  $$f(u)=\alpha \beta \sigma u^{\alpha -1}e^{-\sigma u^{\alpha }}{\left(1-e^{-\sigma u^{\alpha }}\right)}^{\beta },\;\;\;\;\;\;\;\;u>0,\alpha ,\beta ,\sigma >0.$$
• MO-Weibull distribution
  $$f(u)=\frac{\alpha \sigma u^{\alpha -1}e^{-\sigma u^{\alpha }}}{{\left(1-(1-\theta )e^{-\sigma u^{\alpha }}\right)}^{2}},\;\;\;\;\;\;\;\;u>0,\alpha ,\theta ,\sigma >0.$$
• APT-Weibull distribution
  $$f(u)=\frac{\log\left(\alpha_{1}\right)\alpha \sigma u^{\alpha -1}e^{-\sigma u^{\alpha }}}{\alpha_{1}-1}\alpha_{1}^{\left(1-e^{-\sigma u^{\alpha }}\right)},\;\;\;\;\;\;\;\;u>0,\alpha ,\alpha_{1},\sigma >0.$$

### 6.3 Decisive tools

In this subsection, we discuss some decisive tools (i.e., selection criteria) to prove which probability distribution offers the best fit to the flood data set. The decisive tools are

• AIC
  2k−2ℓ.
• CAIC
  $$\frac{2nk}{n-k-1}-2\ell .$$
• BIC
  klog⁡(n)−2ℓ.
• HQIC
  2klog⁡(log⁡(n))−2ℓ.
• The AD test
  $$-\frac{1}{n}\sum_{i=1}^{n}\left(2i-1\right)\left[\log F\left(u_{i}\right)+\log\left\{1-F\left(u_{n-i+1}\right)\right\}\right]-n.$$
• The CM test
  $$\sum_{i=1}^{n}{\left[\frac{2i-1}{2n}-F\left(u_{i}\right)\right]}^{2}+\frac{1}{12n}.$$
• The KS test statistic
  $$KS=sup_{u}\left[F_{n}\left(u\right)-F\left(u\right)\right].$$

Besides the above decisive tools, the *p-value* associated with the KS statistic is also calculated. It is a very important statistical measure that indicates the efficiency of the competing models. The higher *p-value* represents the higher efficiency of the model, in other words, the model with the highest *p-value*, provides the best fit (or adequate or reasonable fit) to the data.

### 6.4 Analysis of the flood data

We apply the NFWE distribution and its competing distributions to analyze flood data. The results of these distributions for the flood data set are prevailed by utilizing the R-script with optim() and method=“BFGS”; see Appendix B.

After performing the analysis using the flood data set, Table 9 presents the estimated parameters values. Whereas, the values of the decisive tools are presented in Table 10, Table 11. For the NFWE distribution, the p-value shows the highest value and the fitting criteria shows the lowest numerical values. These facts provide concrete evidence that the NFWE distribution is the best model for the flood data.

**Table 9:** The values of $\overset{ˆ}{\delta_{1}}$, $\overset{ˆ}{\delta_{2}}$, $\overset{ˆ}{\alpha }$, $\overset{ˆ}{\sigma }$, $\overset{ˆ}{\beta }$, $\overset{ˆ}{\theta }$, and $\overset{ˆ}{\alpha_{1}}$ using the flood data.

Model	$\overset{ˆ}{\delta_{1}}$	$\overset{ˆ}{\delta_{2}}$	$\overset{ˆ}{\alpha }$	$\overset{ˆ}{\sigma }$	$\overset{ˆ}{\beta }$	$\overset{ˆ}{\theta }$	$\overset{ˆ}{\alpha_{1}}$
NFWE	0.25316 (0.02914)	1.20345 (0.08620)	-	-	-	-	-
FWE	0.30851 (0.02441)	0.89727 (0.07936)	-	-	-	-	-
Weibull	-	-	1.28964 (0.08034)	0.54393 (0.05892)	-	-	-
EW	-	-	0.33082 (0.10770)	4.38781 (1.51141)	57.69328 (5.17422)	-	-
MOW	-	-	1.92717 (0.18435)	0.07618 (0.05317)	-	0.09086 (0.06973)	-
APTW	-	-	1.53394 (0.09846)	0.22445 (0.07192)	-	-	0.07538 (0.07443)

**Table 10:** The values of the fitting criteria using the flood data.

Model	AIC	CAIC	BIC	HQIC
NFWE	345.58160	345.66860	351.47910	347.97810
FWE	353.07870	353.16560	358.97620	355.47520
Weibull	380.40110	380.48810	386.29860	382.79770
EW	357.53520	357.71040	366.38150	361.13000
MOW	365.59290	365.76800	374.43910	369.18770
APTW	374.28170	374.45690	383.12800	377.87650

**Table 11:** The values of the fitting criteria using the flood data.

Model	CM	AD	KS	*p-value*
NFWE	0.04170	0.25790	0.04100	0.97150
FWE	0.10970	0.71910	0.10500	0.08910
Weibull	0.44160	2.69510	0.11250	0.06500
EW	0.12150	0.75310	0.06460	0.59720
MOW	0.22950	1.39780	0.08080	0.31480
APTW	0.34370	2.09210	0.10210	0.10570

**Figure 4:**
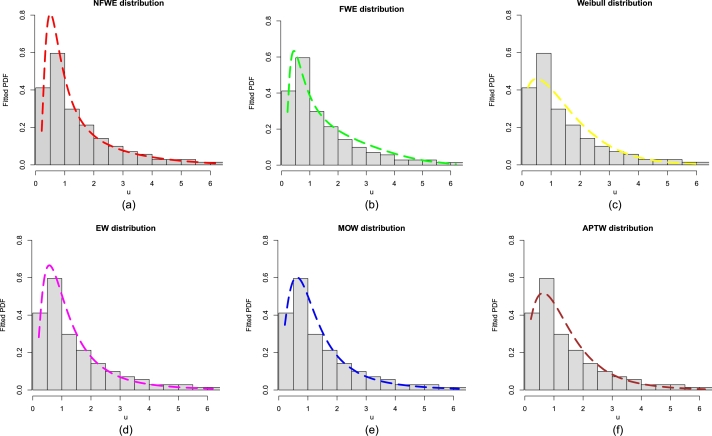
The estimated PDFs of (a) NFWE, (b) FWE, (c) Weibull distribution, (d) EW, (e) MOW, and (f) APTW distribution for the flood data.

**Figure 5:**
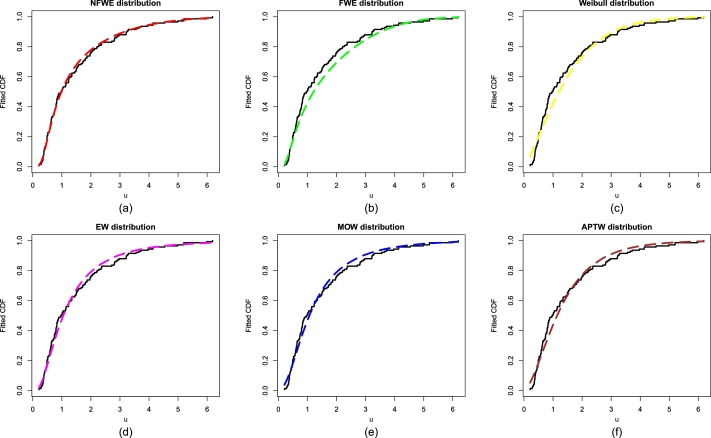
The estimated CDFs of (a) NFWE, (b) FWE, (c) Weibull distribution, (d) EW, (e) MOW, and (f) APTW distribution for the flood data.

The best fitting claim of the NFWE distribution in Table 10, Table 11, is also supported visually in Fig. 4(a-f) and Fig. 5(a-f). The plots in Fig. 4(a-f) and Fig. 5(a-f) also confirm the suitability of the NFWE distribution for the flood data.

## 7 Final remarks

This paper considered a NFWE distribution as a potential modification of the FWE model using the weighted T-*X* procedure. For the NFWE model, some distributional characteristics were derived. The estimators of the FWE distribution were obtained through different estimation methods. For the NFWE distribution, the best estimation method was identified using a comprehensive simulation study. The practical evaluation of the NFWE distribution was demonstrated by considering the flood data with extreme observations. By deciding on different comparative tests, there were concrete evidence that the NFWE distribution is the most appropriate model (i.e., an adequate competing model) for the flood and other extreme event data.

The future work includes the development of (i) bivariate and multivariate modifications of the NFWE model and their practical implementations, (ii) regression structure of the NFWE model for survival data, and (iii) neutrosophic extension of the NFWE model for environmental studies.

## CRediT authorship contribution statement

**Huda M. Alshanbari:** Conceptualization, Data curation, Formal analysis, Investigation, Methodology, Software, Validation, Visualization, Writing – original draft, Writing – review & editing. **Omalsad Hamood Odhah:** Conceptualization, Data curation, Formal analysis, Investigation, Methodology, Software, Validation, Visualization, Writing – original draft, Writing – review & editing. **Hazem Al-Mofleh:** Conceptualization, Data curation, Formal analysis, Investigation, Methodology, Software, Validation, Visualization, Writing – original draft, Writing – review & editing. **Zubair Ahmad:** Conceptualization, Data curation, Formal analysis, Investigation, Methodology, Software, Validation, Visualization, Writing – original draft, Writing – review & editing. **Saima K. Khosa:** Conceptualization, Data curation, Formal analysis, Investigation, Methodology, Software, Validation, Visualization, Writing – original draft, Writing – review & editing. **Abd al-Aziz Hosni El-Bagoury:** Conceptualization, Data curation, Formal analysis, Investigation, Methodology, Software, Validation, Visualization, Writing – original draft, Writing – review & editing.

## Declaration of Competing Interest

The authors declare no conflict of interest.

## Data Availability

Data included in article/supplementary material/referenced in article.

## Appendix A

The observations of the flood data set are: 0.7791985, 1.1483097, 2.9672206, 1.4938204, 1.5833982, 1.7602758, 0.2158896, 0.3316141, 0.9555678, 0.6043510, 0.8510060, 0.4920070, 0.3715561, 0.6607851, 0.4876386, 0.4834433, 0.7375517, 0.5111949, 1.1339544, 0.3851595, 2.3691135, 0.5186865, 0.4137324, 0.7189331, 0.7381207, 0.3792905, 0.3972784, 0.6167996, 0.8184564, 0.3787097, 2.0782179, 1.4738027, 2.2733590, 1.4995140, 2.3685689, 0.7929649, 1.3452230, 3.7218931, 0.4847362, 0.5570308, 0.6421779, 0.3521637, 1.2319488, 1.0085401, 0.5739284, 0.8282354, 1.3438912, 0.7985639, 0.5913791, 0.3593687, 0.6421779, 0.3521637, 1.2319488, 1.0085401, 0.5739284, 0.8282354, 1.3438912, 0.7985639, 0.5913791, 0.3593687, 1.2470297, 1.5119436, 1.2313264, 0.4174427, 0.4856877, 0.7467844, 1.4316524, 2.0115124, 0.5920552, 0.6354470, 0.2082874, 0.8970404, 0.4521464, 0.8233669, 1.0459246, 0.8731764, 0.8302978, 1.5595648, 0.8808601, 1.5319633, 0.7943833, 1.0328066, 2.1259828, 0.6339371, 1.3113389, 0.8507588, 1.1216619, 4.5418208, 1.2421274, 3.5577773, 2.0439183, 0.3183116, 0.7603422, 0.6969910, 0.6208522, 0.7419367, 2.3501271, 0.4923549, 0.9713638, 1.7159552, 1.1431615, 0.5176597, 0.6126890, 0.3778499, 0.4487731, 0.4412745, 0.2943709, 0.2843115, 0.3939066, 0.4730012, 1.8675487, 1.6653246, 1.9875657, 1.7238783, 1.8093874, 1.9698237, 1.8123847, 1.9654765, 1.8654765, 2.1987234, 2.2165476, 2.8654765, 2.9216547, 2.8012938, 2.8092834, 2.7165476, 2.8165476, 3.2654765, 3.2912873, 3.2091827, 3.2234893, 3.6547652, 3.9654765, 3.2187324, 4.9827360, 4.1372098, 4.1412307, 5.1987322, 5.1988294, 6.1876232, 5.9826342.

## Appendix B

### B.1 R code for calculating the MLEs and goodness-of-fit measures

***Note:*** In the following R-code, del1 is used for $\delta_{1}$, del2 is used for $\delta_{2}$, and the variable x is instead of u.

data=c(0.7791985, 1.1483097, 2.9672206, 1.4938204, 1.5833982, 1.7602758, 0.2158896, 0.3316141, 0.9555678, 0.6043510, 0.8510060, 0.4920070, 0.3715561, 0.6607851, 0.4876386, 0.4834433, 0.7375517, 0.5111949, 1.1339544, 0.3851595, 2.3691135, 0.5186865, 0.4137324, 0.7189331, 0.7381207, 0.3792905, 0.3972784, 0.6167996, 0.8184564, 0.3787097, 2.0782179, 1.4738027, 2.2733590, 1.4995140, 2.3685689, 0.7929649, 1.3452230, 3.7218931, 0.4847362, 0.5570308, 0.6421779, 0.3521637, 1.2319488, 1.0085401, 0.5739284, 0.8282354, 1.3438912, 0.7985639, 0.5913791, 0.3593687, 0.6421779, 0.3521637, 1.2319488, 1.0085401, 0.5739284, 0.8282354, 1.3438912, 0.7985639, 0.5913791, 0.3593687, 1.2470297, 1.5119436, 1.2313264, 0.4174427, 0.4856877, 0.7467844, 1.4316524, 2.0115124, 0.5920552, 0.6354470, 0.2082874, 0.8970404, 0.4521464, 0.8233669, 1.0459246, 0.8731764, 0.8302978, 1.5595648, 0.8808601, 1.5319633, 0.7943833, 1.0328066, 2.1259828, 0.6339371, 1.3113389, 0.8507588, 1.1216619, 4.5418208, 1.2421274, 3.5577773, 2.0439183, 0.3183116, 0.7603422, 0.6969910, 0.6208522, 0.7419367, 2.3501271, 0.4923549, 0.9713638, 1.7159552, 1.1431615, 0.5176597, 0.6126890, 0.3778499, 0.4487731, 0.4412745, 0.2943709, 0.2843115, 0.3939066, 0.4730012, 1.8675487, 1.6653246, 1.9875657, 1.7238783, 1.8093874, 1.9698237, 1.8123847, 1.9654765, 1.8654765, 2.1987234, 2.2165476, 2.8654765, 2.9216547, 2.8012938, 2.8092834, 2.7165476, 2.8165476, 3.2654765, 3.2912873, 3.2091827, 3.2234893, 3.6547652, 3.9654765, 3.2187324, 4.9827360, 4.1372098, 4.1412307, 5.1987322, 5.1988294, 6.1876232, 5.9826342) #---PDF pdf_pm <- function(par,x) { del1= par[1] del2= par[2] (del1+(del2/x^2))*exp((del1*x)-(del2/x))*exp(-exp((del1*x)-(del2/x))) *(1+exp(-exp((del1*x)-(del2/x))))*(1/(exp(1-exp(-exp((del1*x)-(del2/x)))))) } #---CDF cdf_pm <- function(par,x) { del1= par[1] del2= par[2] 1-((exp(-exp((del1*x)-(del2/x))))/(exp(1-exp(-exp((del1*x)-(del2/x)))))) } set.seed(0) goodness.fit(pdf=pdf_pm, cdf=cdf_pm, starts = c(0.5,0.5), data = data, method="BFGS", domain=c(0,Inf),mle=NULL)

### B.2 R code for calculating the MPS estimators

S=function(para, x,cdf0,pdf0) { n=length(x) x=sort(x) D = rep(0,l=(n+1)) D[1]= cdf0(x[1],para) D[n+1] = 1-cdf0(x[n],para) for(i in 2:n){ if(x[i]==x[i-1]){ D[i] = pdf0(x[i],para) }else{ D[i] = cdf0(x[i],para)-cdf0(x[i-1],para) } } -(1/(n+1))*sum(log(D)) } MPS = function (cdf0,pdf0,theta0,data,lim_inf,lim_sup) { max = nlminb(start = theta0, objective=S,x=data,cdf0=cdf0, pdf0=pdf0,lower = lim_inf, upper = lim_sup, control = list(iter.max=500,rel.tol=1e-6)) return(max$par) }
